# Electron-Withdrawing Effects in Cobalt Porphyrin Catalysts Boost Homogeneous Photocatalytic Hydrogen Evolution in Neutral Aqueous Solutions

**DOI:** 10.1021/acscatal.5c00788

**Published:** 2025-03-05

**Authors:** Chengyu Liu, Titus de Haas, Francesco Buda, Sylvestre Bonnet

**Affiliations:** Leiden Institute of Chemistry, Leiden University, Einsteinweg 55, P.O. Box 9502, 2333CC Leiden, The Netherlands

**Keywords:** homogeneous photocatalysis, hydrogen evolution, cobalt porphyrin, molecular catalyst, DFT calculations

## Abstract

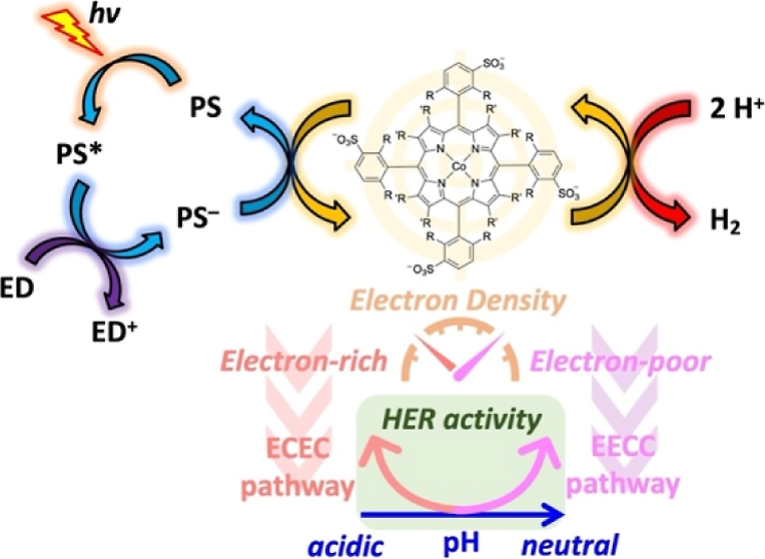

Molecular catalysts offer an ideal platform for conducting mechanistic studies of the hydrogen evolution reaction (HER) due to their electronic tunability. This study explores a series of anionic M=Co(III)- and M=Zn(II)-porphyrin complexes with electron-donating ([M(OMeP)]^*n*−^, [M(MeP)]^*n*−^) and electron-withdrawing ([M(F8P)]^*n*−^, [M(F16P)]^*n*−^) substituents. The activity of these complexes for the HER was analyzed in homogeneous photocatalytic conditions using [Ru(bpy)_3_]^2+^ as a photosensitizer under blue light (450 nm) irradiation. The substituent-induced electronic effects were found to tightly control the activity and mechanism of the photocatalytic HER. As expected, the electron-rich [Co(OMeP)]^3–^ catalyst showed higher activity in acidic media (pH 4.1) with a maximum TOF of 7.2 ± 0.4 h^–1^ and TON of 175 ± 5 after 39.5 h. DFT calculations were performed to investigate the HER mechanism. H_2_ formation was found to initiate following proton-coupled reduction of a Co^III^–H hydride intermediate in such conditions. More surprisingly, however, the electron-poor [Co(F16P)]^3–^ catalyst was more active at neutral pH (7.0), achieving a maximum TOF of 6.7 ± 0.3 h^–1^ and TON of 70 ± 3 after 39.5 h. Instead of forming the Co^III^–H hydride, an additional ligand-based reduction led to a ligand-protonated intermediate. This work demonstrates that electron-poor HER catalysts can outperform electron-rich catalysts near neutral pH conditions.

## 1 Introduction

Light-induced H_2_ evolution in aqueous solutions has received great attention since it could contribute to providing a sustainable and environmentally friendly energy conversion system.^1^ In order to drive this photoreaction efficiently, well-performing hydrogen-evolving catalysts (HECs) must be prepared, the design of which is not yet fully understood.^2−4^ Recently, studies on molecular hydrogen evolution catalysts have multiplied due to their numerous advantages: (1) they mimic the active sites of heterogeneous catalysts and allow to approach catalytic mechanisms with atomic precision;^5−7^ (2) ligand functionalization allows for fine-tuning the coordination sphere and electronic density of the metal center, which is a powerful tool to establish relationships between the properties of the metal center and its photocatalytic performances;^7,8^ and (3) they can be integrated in supramolecular water-splitting systems, also in combination with solid-state materials and catalysts.^9−11^

In the past decade, molecular HECs have been extensively studied for photocatalytic hydrogen evolution, especially those made of first-row transition metals.^12−28^ Many of those catalysts are based on cobalt, which is particularly attractive as it is available in larger quantities on Earth than platinum or ruthenium, and because it has more than five available oxidation states.^29,30^ Usually, these catalysts show better catalytic properties in mildly acidic conditions (pH 4–5), where more protons are available for the fast generation of H_2_. On the other hand, coupling proton reduction and water oxidation to achieve a full water splitting scheme, may be more favorable in neutral conditions, because water oxidation becomes more challenging at acidic pH. Still, examples of cobalt-based HECs working at pH 7.0 in homogeneous aqueous solutions are very rare.^31,32^

**Figure 1:**
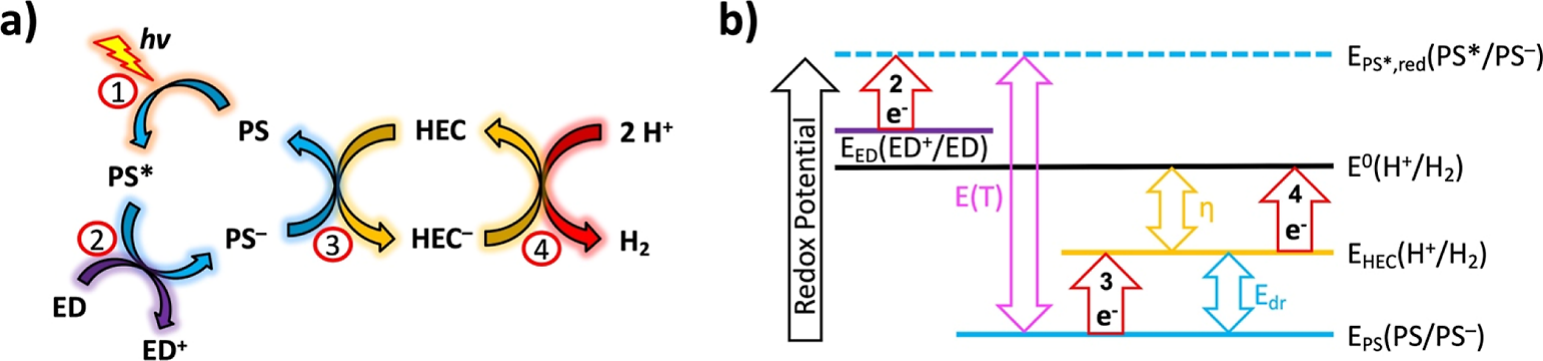
(a) Simplified photocatalytic mechanism and (b) relation between the redox potentials (in V) of different redox couples of the reductive quenching pathway in a three-component molecular photocatalytic hydrogen-evolution system. ED: sacrificial electron donor; PS: photosensitizer; HEC: hydrogen evolution catalyst; *E*(T): triplet excited state energy of the PS; η: overpotential of the catalyst; *E*_dr_: driving force (in V) of the electron-transfer step from the reduced photosensitizer PS^–^ to the catalyst (see text for exact definition).

Photocatalytic H_2_-evolving systems contain, next to the catalyst, a photosensitizer (PS) and a sacrificial electron donor (ED). In such 3-component systems the much higher concentration of the ED, compared to the hydrogen evolution catalyst (HEC), usually favors kinetically the excited state PS* to be quenched reductively by ED to afford PS^–^ (step 2 in Figure 1a).^4^ PS^–^ then reduces HEC to HEC^–^ (step 3), an electron-transfer step driven by the driving force *E*_dr_ (in V), defined as the difference between the redox potential of the PS/PS^–^ couple and the catalytic onset potential, itself defined as the potential at which electrocatalytic H_2_ evolution catalyzed by HEC becomes significant. This process, when repeated twice, leads to the formation of an H_2_ molecule (step 4). In such a system a good balance between *E*_dr_ and the overpotential of the catalyst (η) is needed to keep both step 3 and step 4 fast.^8^ Functionalization of the catalyst with electron-donating or electron-withdrawing groups in principle allows for optimization of *E*_dr_ and η. However, for synthetic reasons it is not always easy to vary systematically the redox properties of a cobalt complex to optimize its redox potential for catalytic hydrogen reduction.^19,33^

Porphyrin ligands offer stable and rigid coordination sites for metal centers, while being readily tunable with functional substituents, which makes cobalt porphyrin a very attractive compound for HEC development.^1,33−35^ Importantly, two hydrogen evolution mechanisms for step 4 (Figure 1a) have been reported with cobalt porphyrin catalysts.^36^ In the first one, initial electroreduction of the Co^II^ center to Co^I^ is followed directly by a chemical oxidative protonation (EC mechanism), forming a Co^III^ hydride (Co^III^–H).^37−40^ The second electrochemical reduction step then leads to fast formation of dihydrogen via the protonolysis pathway. Weak acids, on the other hand, typically do not protonate the Co^I^ metal center and a second reduction step is required before oxidative protonation and subsequent protonolysis can occur.^35−38,41^ Similar behavior has been observed for copper porphyrins and aminopyridine cobalt complexes.^42,43^ The first mechanism is referred to as an ECEC mechanism, while the second is usually denoted as EECC. As the formally Co^I^/Co^0^ couple has been shown to be partially ligand based, the EECC mechanism can involve catalysis on the ligand rather than on the metal center.^37^ Therefore, the activity of a cobalt catalyst (step 4 in Figure 1a) may depend strongly on the pH, and the different mechanisms may be contingent on the electron-density on the metal catalyst. In general, it remains poorly understood which influence electron-withdrawing or electron-donating groups have on the (photo)catalytic activity of HEC, and in particular on the pH dependence.

**Figure 2:**
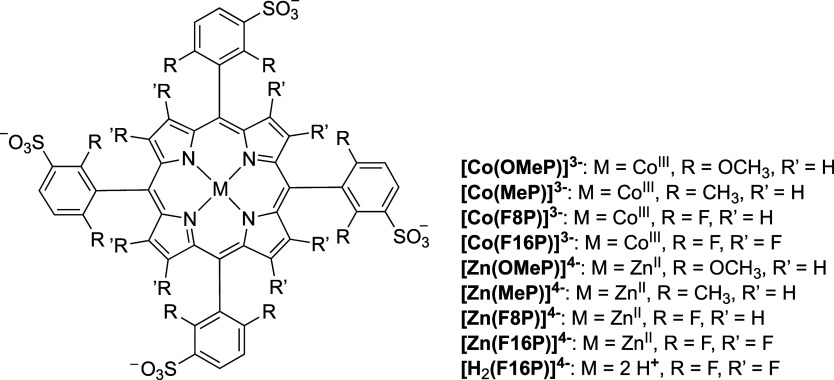
Chemical structures of the water-soluble metal porphyrin complexes and free base ligands used in this work. All compounds were isolated as Na^+^ salts.

To bridge this knowledge gap, in this work a family of sulfonated, water-soluble Co(III)-porphyrin complexes was prepared bearing either electron-donating ([Co(OMeP)]^3–^, [Co(MeP)]^3–^) or electron-withdrawing ([Co(F8P)]^3–^, [Co(F16P)]^3–^) substituents (Figure 2). This series of compounds was used to investigate the effect of the electron density of the ligand on homogeneous photocatalytic hydrogen evolution in the presence of [Ru(bpy)_3_]Cl_2_ as photosensitizer (PS), and ascorbic acid (AA) in combination with tris(2-carboxyethyl)phosphine hydrochloride (TCEP) as sacrificial electron donor (ED). Using TCEP as final electron donor allows ascorbate to be recycled, enabling it to function as a reversible electron relay. This additional donor is known to significantly inhibits electron back transfer from PS^–^ to the ascorbate radical, sometimes leading to much higher turnover numbers in photocatalytic hydrogen evolution compared with using ascorbate only.^12,44^ The pH dependence of the (photo)catalytic properties of these systems was investigated and compared to those of the Zn^2+^ analogues, in which the metal center cannot accommodate any changes of the oxidation state, while the ligand remains potentially redox active. The mechanism of cobalt-porphyrin catalyzed hydrogen evolution is here clarified with DFT calculations, which show a change from an ECEC to an EECC mechanism by reducing the electron richness of the cobalt center. Consequently, the electron-poor catalyst favors a ligand-based mechanism over a metal-centered mechanism, resulting in increased catalytic activity in higher pH, highlighting a new design principle for proton reduction catalysts for solar-to-fuel devices.

## 2 Results and Discussion

### 2.1 Synthesis

The tetra-sulfonated, free-base porphyrin ligands Na_4_[H_2_(OMeP)],^8^ Na_4_[H_2_(MeP)],^45^ Na_4_[H_2_(F8P)],^46^ Na_4_[H_2_(F16P)],^47^ and their metal complexes Na_3_[Co(F8P)],^48,49^ Na_4_[Zn(F8P)],^50^ Na_4_[Zn(F16P)],^47^ were synthesized according to reported methods. The other Co(III) and Zn(II) porphyrin compounds described in this work are new and were synthesized via refluxing the free-base ligands with Co(II) sulfate or Zn(II) dichloride in Milli-Q water for 12 h. Na^+^-loaded ion-exchange resin was used to exchange the counterions with Na^+^, and the complexes were purified by size-exclusion chromatography in order to remove excess inorganic salts. According to NMR all cobalt compounds were diamagnetic and hence contained low-spin Co(III) ions. The Co(III) porphyrin complexes were formed by aerial oxidation of Co(II) during purification.^48,49,51^ Full characterization is given in the Supporting Information (Figures S1–S6).

### 2.2 Electrochemical Properties

The electron-richness of a ligand influences the redox and electrocatalytic properties of its metal complex.^7,32^ A classical idea in hydrogen-evolution catalysis is that lower pH values render most catalysts more efficient at catalyzing the HER. The redox properties of the Co(III)- and Zn(II)-porphyrin complexes were hence studied with electrochemical methods in pH 7.0 and pH 4.1 aqueous solutions and *N*,*N*-dimethylformamide (DMF) solution. Cyclic voltammetry (CV), linear sweep voltammetry (LSV), and differential pulse voltammetry (DPV), were recorded using a glassy-carbon (GC) working electrode in an aqueous phosphate buffer or a tetrabutylammonium hexafluorophosphate (TBAPF_6_) DMF solution.

**Figure 3:**
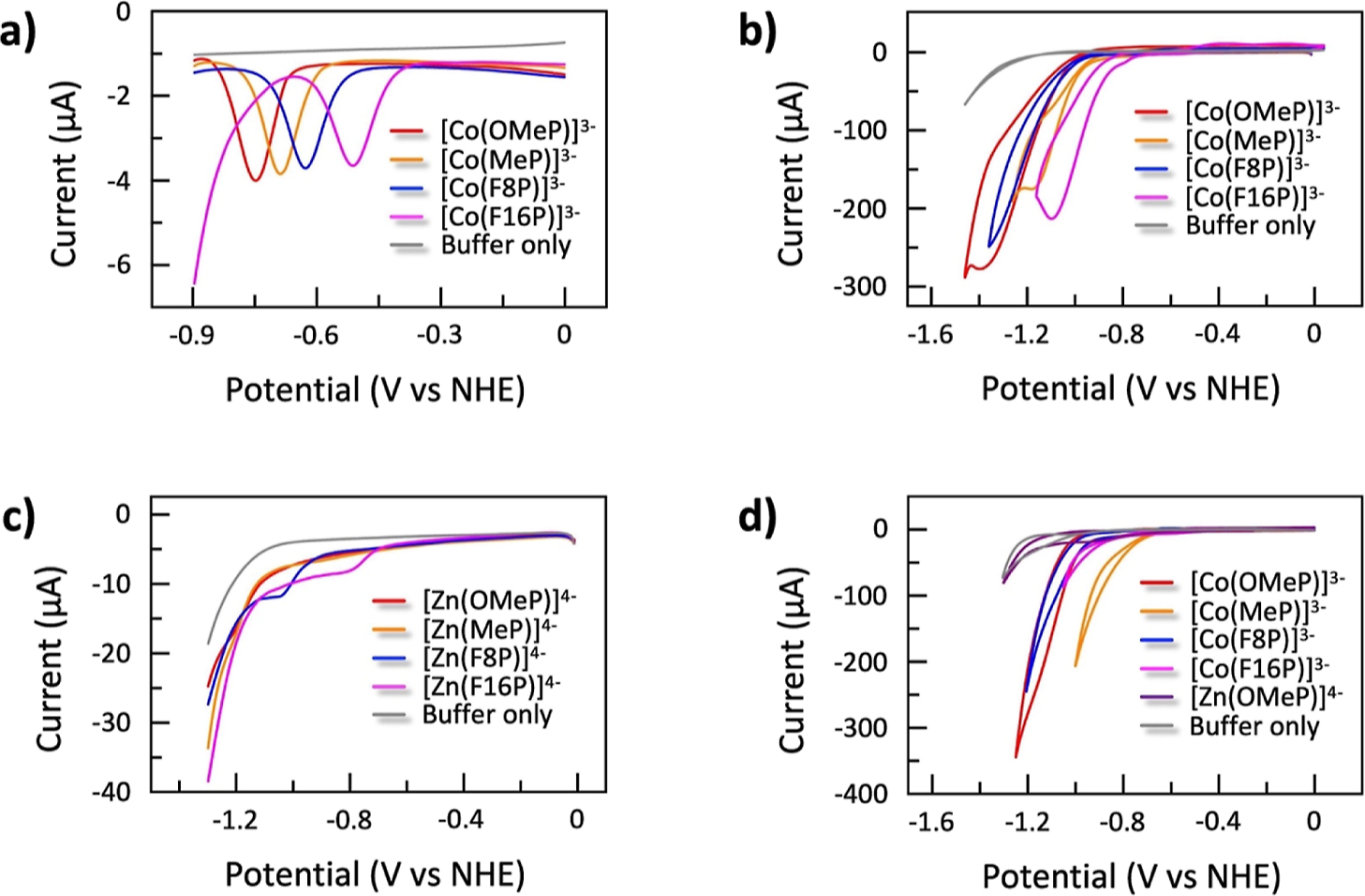
Electrochemistry at pH 7.0 and 4.1 of the water-soluble metal porphyrin complexes studied in this work. (a) Differential pulse voltammetry (DPV) and (b) cyclic voltammetry (CV) of the Co-porphyrin complexes at pH 7.0; (c) linear sweep voltammetry (LSV) of the Zn porphyrin complexes at pH 7.0 and (d) CV of the Co-porphyrin complexes at pH 4.1. Conditions: 0.1 M phosphate buffer for pH 7.0 and 1.0 M phosphate buffer for pH 4.1, 0.07 cm^2^ glassy-carbon working electrode, Pt wire auxiliary electrode, Ag/AgCl reference electrode, CV and LSV scan rate 50 mV s^–1^, *T* = 298 K. DPV experimental parameters: 0.004 V increase potential, 0.05 V amplitude, 0.05 s pulse width, 0.0167 s sampling width, 0.5 s sample period.

The initial reduction of Co(III)-porphyrin (Co^III^(P)) complexes to Co(II)-porphyrin (Co^II^(P)) takes place at positive potentials vs NHE. While this reduction was difficult to detect in aqueous solution, a distinct reduction wave was clearly observable in DMF. This behavior is illustrated in Figure S7a, which presents the CV of [Co(F8P)]^3–^ in DMF with increasing water content. Based on this analysis, after the first reduction, the DPV traces of the four cobalt porphyrin complexes at pH 7.0 (Figure 3a) are assigned to reduction of the Co^II^(P) to Co(I)-porphyrin (Co^I^(P)). Lowering the electron density of the complex led to a more negative potential of this second reduction wave, which was observed at −0.53 V vs NHE for the most electron-poor complex [Co(F16P)]^3–^ and at −0.75 V vs NHE for the most electron-rich compound [Co(OMeP)]^3–^. Figure S8 in the ESI demonstrates that a 1-electron process induces a current change of −8 μA. Based on this, a catalytic onset is defined as a current increase exceeding −16 μA, corresponding to a process involving more than two electrons. Cyclic voltammograms recorded at pH 7.0 revealed that the third reduction of the cobalt porphyrin complexes induced electrocatalytic hydrogen evolution with an onset potential between −0.84 and −1.04 V vs NHE depending on the substituents (Figure 3b). Interestingly, the trend in the variation of the third reduction potential does not align with the electron-richness of the cobalt center, as the onset potential for the [Co(MeP)]^3–^ catalyst was found to be more positive than that found for the [Co(F8P)]^3–^ catalyst. This observation suggested that the HER catalytic mechanism may have changed when switching from an electron-rich [Co(MeP)]^3–^ to an electron-poor [Co(F8P)]^3–^ catalyst. A control CV measurement was conducted using [Co(F8P)]^3–^ in 0.1 M KCl (Figure S7b). The reduction of Co(II) to Co(I) occurred at −0.62 V vs NHE, and the reduction of Co(I) to Co(0) was observed at −1.06 V vs NHE. These values closely correspond to −0.64 and −1.01 V vs NHE, respectively, measured in a pH 7.0 buffer. As a conclusion, the phosphate buffer did not significantly affect the potentials of the catalytic waves for this complex, hence our data in buffer solution can be used for comparison with DFT calculations (see below). The redox potential of the [Ru(bpy)_3_]^2+^/[Ru(bpy)_3_]^+^ couple lies between −1.26 and −1.32 V vs NHE depending on the reports.^13,52,53^ Therefore, the one-electron reduced species PS^–^ is thermodynamically capable of driving the reduction of all four Co(III)–porphyrin compounds to a potential where catalytic HER occurs, with an electron-transfer driving force *E*_dr_ (Figure 1b) exceeding 160 mV. It is worth noting that for [Co(F16P)]^3–^ a small reduction wave was observed at −0.80 V vs NHE at the foot of the catalytic wave (Figure 3b), which is attributed to a ligand-based reduction, as at a similar potential, a reduction wave was observed for the [Zn(F16P)]^4–^ analogue, for which metal-based reduction is unlikely (Figure 3c).

The LSV traces of the Zn porphyrin complexes were measured at pH 7.0, focusing on reduction. As expected, the more electron-poor [Zn(F16P)]^4–^ showed the least negative reduction potential (−0.80 V vs NHE, see Figure 3c). There is little increase in reduction current with more negative potential until −1.10 V vs NHE, which suggested that this process concerns a reductive polarization of the complex without catalysis. In addition, the reduction current was approximately −8 μA, which was similar to the current of the 1e^–^ reduction of Co^II^ to Co^I^ (−8 μA, see Figure S8). Thus, this first reduction of this Zn porphyrin most likely involves 1 electron. For [Zn(F8P)]^4–^, more than one but less than two electrons were needed to form the species active for the further HER, which took place at −1.20 V vs NHE. Reduction of the more electron-rich [Zn(MeP)]^4–^ and [Zn(OMeP)]^4–^ was only observed below −1.20 V vs NHE with reduction currents higher than −16 μA; these two compounds hence underwent a reduction process involving more than 2 electrons (Figure S9). Overall, we tentatively ascribe this process to electrocatalytic HER. In aqueous solution at pH 7.0, the reduced form of the photosensitizer, [Ru(bpy)_3_]^+^, should also be capable of driving the 2-electron reduction of the Zn-porphyrin complexes and HER catalyzed by the Zn compounds, with a driving force *E*_dr_ that is higher than 60 mV.

At pH 4.1, CVs showed that all cobalt porphyrin complexes were also electrocatalytically active for the HER (Figure 3d). As expected, the compounds [Co(OMeP)]^3–^, [Co(MeP)]^3–^ and [Co(F8P)]^3–^ showed a higher catalytic current at pH 4.1 than at pH 7.0 with the same overpotential. However, the electrocatalytic current obtained with [Co(F16P)]^3–^ was lower at pH 4.1 (<−100 μA) than at pH 7.0 (>−200 μA), with 200 mV overpotential. This result strongly suggested that the most electron-poor catalyst of the series may catalyze the HER at neutral pH more efficiently than at pH 4.1, possibly by following a different mechanism than the other complexes.

### 2.3 Photocatalysis

**Figure 4:**
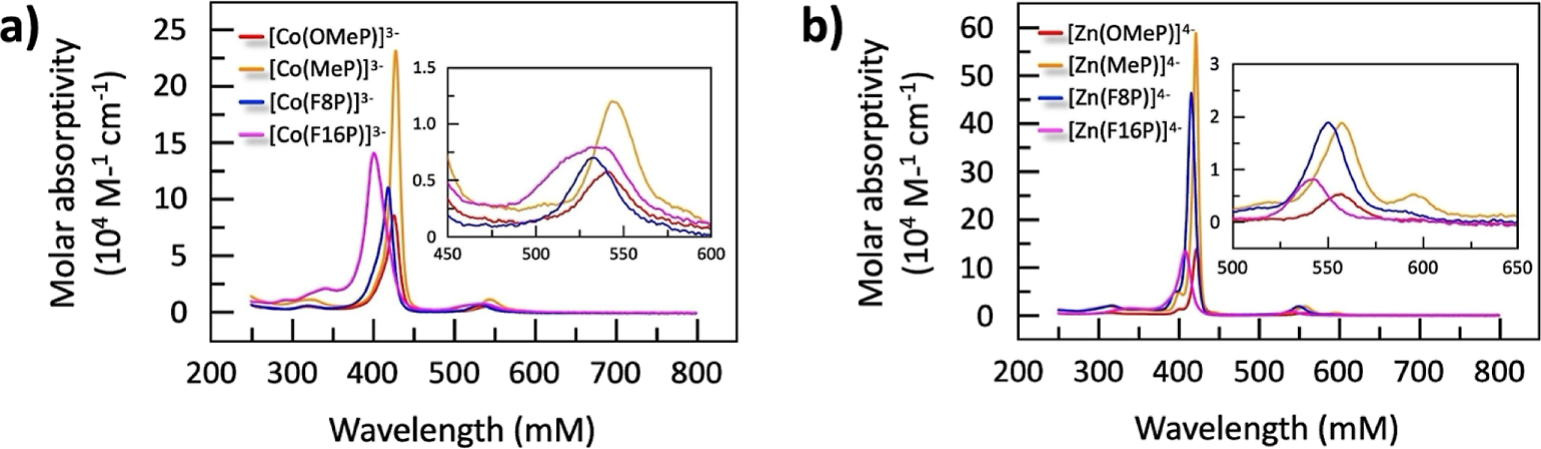
UV–vis spectra of Co(III)- (a) and Zn(II)-porphyrins (b) used in this work.

**Figure 5:**
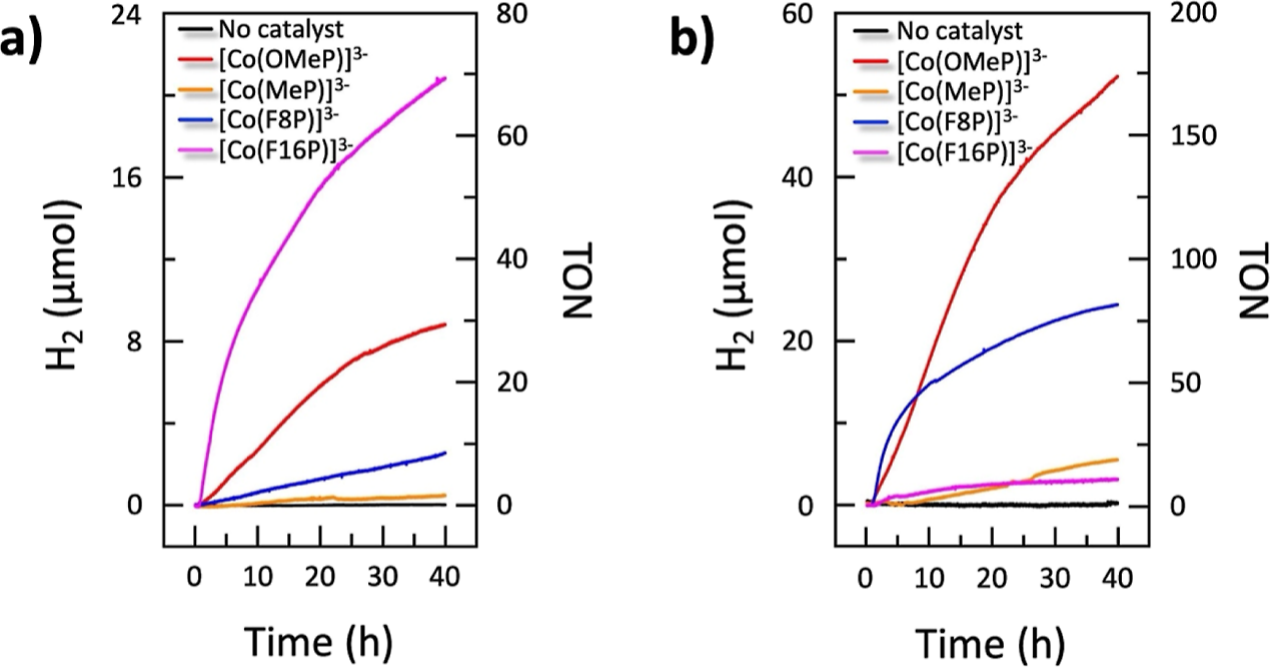
Hydrogen evolution during photocatalytic water reduction in homogeneous aqueous solution in the presence of 0.1 mM catalyst [Co(OMeP)]^3–^, [Co(MeP)]^3–^, [Co(F8P)]^3–^ or [Co(F16P)]^3–^, 0.5 mM [Ru(bpy)_3_]Cl_2_ as photosensitizer, 0.1 M ascorbate and TCEP as electron donor, at pH 7.0 (a) or 4.1 (b), using blue light irradiation (450 nm, 16 mW). Conditions: *T* = 298 K, 0.1 M phosphate aqueous buffer.

Considering the encouraging electrocatalytic results described above, the photocatalytic activity of all metal porphyrin complexes in HER was tested in homogeneous aqueous solutions using [Ru(bpy)_3_]Cl_2_ as PS (0.5 mM), AA and TCEP (100 mM each) as sacrificial electron donors, and under blue light irradiation (450 nm, 16 mW). Importantly, all these cobalt and zinc porphyrin complexes have no significant absorption at 450 nm (Figure 4), therefore, negligible photons would be absorbed by the catalyst instead of the photosensitizer during photocatalysis. Unlike in electrocatalytic conditions where all cobalt porphyrins showed catalytic activity for proton reduction, in photocatalytic conditions at pH 7.0 only the most electron-poor and the most electron-rich complexes showed significant HER activity (Figure 5a). In contrast, the most electron-rich complex [Co(OMeP)]^3–^ had a lower activity than the electron-poor complex [Co(F16P)]^3–^, which showed the highest turnover number in the series (TON = 70 ± 3 mol_H_2__/mol_HEC_ after 39.5 h irradiation) and the highest maximum turnover frequency (TOF = 6.7 ± 0.3 h^–1^, defined in the “Materials and Methods” section). At that stage, it was surprising to find [Co(F16P)]^3–^ so active at pH 7.0. Although this complex had the highest driving force for electron transfer *E*_dr_ (Figure 1b) in the four cobalt porphyrins according to their onset potentials (Table S1), this was not the key reason for its high performance, as [Co(MeP)]^3–^ had a *E*_dr_ 90 mV stronger than that of [Co(OMeP)]^3–^ while [Co(MeP)]^3–^ showed no better photocatalytic HER activity than [Co(OMeP)]^3–^ under such conditions.

At pH 4.1, the photocatalytic HER activity of electron-rich compounds [Co(OMeP)]^3–^ and [Co(MeP)]^3–^ as well as the electron-poor compound [Co(F8P)]^3–^, all improved compared with pH 7.0 (Figure 5b). After 39.5 h irradiation, [Co(OMeP)]^3–^ showed the highest TON of the series (175 ± 5 mol_H_2__/mol_HEC_ after 39.5 h) and a maximum TOF of 7.2 ± 0.4 TON/h. By contrast, [Co(MeP)]^3–^ was not active at pH 7.0 but showed at pH 4.1 a TON of 22. [Co(F8P)]^3–^ showed the fastest turnover frequency at the beginning of the reaction (12.7 h^–1^), but the system was not stable, resulting in a TON of only 80 after 39.5 h. In contrast, the electron-poorest complex [Co(F16P)]^3–^ showed a significantly lower photocatalytic activity at pH 4.1 (Figure 5b), characterized by a much lower TON = 10 after 39.5 h irradiation, compared to pH 7.0 (TON = 70).

All together, these photocatalytic results are consistent with those observed in electrochemical conditions. The electron-withdrawing fluorine substituents appear to influence the electron density at the metal center and change the pH dependence of the catalytic activity, which are reversed compared with that of the electron-rich cobalt catalysts. This, in turn, suggested that the mechanism of catalytic proton reduction at the porphyrin complexes were different for the electron-rich and electron-poor cobalt compounds.

Photocatalytic hydrogen evolution using the Zn-porphyrin complexes as catalysts was also investigated using the same conditions as described above. In the zinc complexes, only the ligand may lead to HEC activity, as the metal center is unable to accommodate different redox states. At pH 7.0 the electron-poor Zn porphyrin complex were the only ones to show significant HER activity: [Zn(F16P)]^4–^ reached a TON of 9.5 after 39.5 h irradiation (Figure S10), while [Zn(F8P)]^4–^ showed lower activity (TON = 6.5). This observation confirmed that a ligand-based catalytic pathway for HER is possible with these porphyrins and that catalysis becomes faster when more electron-withdrawing groups are added to the porphyrin ring. As a note, in these pH neutral conditions [Zn(F16P)]^4–^ and its free-base analogue [H_2_(F16P)]^4–^ showed lower photocatalytic activity than [Co(F16P)]^3–^ (Figure S11a). Therefore, the mechanism of HER with [Co(F16P)]^3–^ as catalyst must not only involve the ligand, but also the redox activity of the cobalt center. In contrast, at pH 4.1 [Zn(OMeP)]^4–^ was found to be inactive (Figure S11b), while [Co(OMeP)]^3–^ showed the highest photocatalytic activity of the cobalt compounds. This result suggests that for the most electron-rich cobalt catalyst of this series the high activity in acidic conditions involves mainly the metal center.

[Co(OMeP)]^3–^ and [Co(F16P)]^3–^ gave the highest TON at pH 4.1 and pH 7.0, respectively. Therefore, the influence of pH on the photocatalytic activity of these two complexes was further studied at different pH values between 4.1 and 9.0, using otherwise identical conditions. Upon increasing the pH from 4.1 to 7.0, the TON after 39.5 h for [Co(F16P)]^3–^ increased from 10 to 70, while the TON decreased to 12 upon further increase of the pH to 9.0 (Figures S12a and S13a). For this complex, pH 7.0 thus appears to be optimal for the stability of the photocatalytic system, although a higher maximum TOF of 8.9 TON/h was found at pH 6.0 (Figure S13b). By contrast, the electron-rich complex [Co(OMeP)]^3–^ clearly showed increasing TON and maximum TOF with more acidic pH, culminating in a TON of 175 and a maximum TOF of 7.2 TON/h at pH 4.1 (Figures S12b and S13).

Overall, classical behavior was observed for the electron-richest complex of the series [Co(OMeP)]^3–^, with more efficient H_2_ generation at low pH values. In contrast, the most electron poor [Co(F16P)]^3–^ catalysts performed optimally in pH-neutral solution, where its TON was twice that of [Co(OMeP)]^3–^. Finally, the photocatalytic activity of [Co(F16P)]^3–^ was inhibited both at pH values higher and lower than 7.0.

### 2.4 Photostability

In order to check the photostability of [Co(F16P)]^3–^ and [Co(OMeP)]^3–^, the UV–vis spectra of solutions of complexes in 0.1 M phosphate buffer (pH 7.0) were monitored both in the dark and upon blue light irradiation for 48 h (450 nm, 16 mW). After 48 h irradiation, the UV–vis spectra of [Co(F16P)]^3–^ and [Co(OMeP)]^3–^ had changed only slightly (Figures S14 and S15), in contrast with [Zn(OMeP)]^4–^, which lost 80% of its absorption properties after 48 h of irradiation at 423 nm. In another experiment using [Co(F16P)]^3–^ or [Co(OMeP)]^3–^ as the catalyst, two consecutive photocatalytic runs were performed, adding a fresh batch of PS between the two runs to compensate for possible PS decomposition. A 3–5% lower TON were found after the second photocatalytic run compared to the first (Figure S16). As widely reported, [Ru(bpy)_3_]^2+^ decomposes quickly in photocatalytic aqueous conditions,^13,54^ which is responsible for deactivation of HER photocatalytic systems. However, clearly both electron-rich and electron-poor Co catalysts had not decomposed during the first photocatalytic run. Overall, these results confirm the excellent photostability of both [Co(F16P)]^3–^ and [Co(OMeP)]^3–^ in such conditions.

### 2.5 Kinetic Study

A full kinetic study was performed for the photocatalytic system comprising the catalyst [Co(F16P)]^3–^ at pH 7.0. Starting at standard photocatalytic conditions of 0.1 mM [Co(F16P)]^3–^, 0.5 mM [Ru(bpy)_3_]Cl_2_, 0.1 M ascorbate and TCEP, and a light source set at 450 nm and 16 mW power, the results were studied of systematic variations in the light intensity, the concentrations of the electron donors, the PS, and the catalyst (Figure S17). We observed the maximum rate of hydrogen evolution of the system to plateau when the light power was higher than 10 mW (Figure S17a), when the concentration of ascorbate and TCEP were higher than 0.075 M (Figure S17b), or with a PS concentration higher than 0.25 mM (Figure S17c). However, a first-order dependence of the maximum H_2_ evolution rate was found on catalyst concentration (Figure S17d). These observations suggests that step 1, step 2 and step 3 in Figure 1 can proceed well independent of the photocatalytic reaction rate. The rate-determining step (RDS) of the system appeared to be step 4, i.e., the catalytic H_2_-evolving step. Considering the linear dependence of the H_2_ production rate with catalyst concentration, it appeared that in the conditions investigated here, the rate-determining step of the catalytic cycle involves one molecule of [Co(F16P)]^3–^. Though we can formally not exclude that at higher concentrations a binuclear mechanism may take place, it is hard to obtain such information as we do expect to reach a plateau, like we did for [Co(OMeP)]^3–^ at concentrations higher than 0.05 mM. As the catalyst molecules are heavily negatively charged, they most probably strongly avoid each other in solution. Using 0.1 mM [Co(F16P)]^3–^ in the standard conditions at pH 7.0, the quantum yield for H_2_ evolution was calculated to be 0.10 ± 0.01%. This low quantum yield highlights the occurrence of many charge-recombination events that are characteristic of homogeneous photocatalytic systems.

A similar kinetic study was performed at pH 4.1 using the catalyst [Co(OMeP)]^3–^, for which significantly different results were obtained than for the study on [Co(F16P)]^3–^. A first-order dependence of the maximum H_2_ evolution rate on light power was found (Figure S17a), indicating that the RDS of this catalytic system involved light, and is thus related to step 1 of the photocatalytic mechanism shown in Figure 1a. Step 4 is relatively fast for this electron-rich catalyst at that low pH at concentrations [HEC] > 0.05 mM. At low concentrations of [Co(OMeP)]^3–^, i.e. 0.05 mM or lower (Figure S17d), a first-order dependence of the maximum hydrogen evolution rate on the catalyst concentration was found. This suggests that the catalytic H_2_ evolution involves one molecule of catalyst. The observation that at higher HEC concentrations the photon flux becomes limiting, aligns with results reported by Alberto and co-workers.^55^ Using 0.1 mM [Co(OMeP)]^3–^ in the standard conditions at pH 4.1, the quantum yield for H_2_ evolution was calculated to be 0.12 ± 0.01%, which is very similar to that for [Co(F16P)]^3–^ at pH 7.0.

Overall, the performed kinetic experiments suggest that, under the conditions of our photocatalytic experiments, the RDS for the [Co(F16P)]^3–^ system is the catalytic H_2_-evolving step (step 4 in Figure 1a). In contrast, the photon flux appears to be the RDS for the [Co(OMeP)]^3–^ system (step 1 in Figure 1a). For both systems the RDS appears different from the electron transfer from PS^–^ to the catalyst in the photocatalytic cycle. Most likely the observed pH dependence in the photocatalytic activity of the two systems is linked to variations in their catalytic mechanisms, instead of differences in their driving forces for electron transfer. While the mechanisms of the two investigated catalysts thus appear to be different, their performances in optimized conditions were comparable.

### 2.6 Density Functional Theory Calculations

To get an understanding of the mechanisms responsible for the hydrogen evolution catalysis with these cobalt catalysts, a detailed density functional theory (DFT) study was performed with GGA functionals and implicit solvation models (see Computational Details section). The aim of these calculations was to establish reaction mechanisms and to identify the most probable reaction intermediates. We performed calculations on the [Co(F16P)]^3–^ and [Co(OMe)]^3–^ complexes, as those two catalysts represent the two most extreme cases of electron-poor and electron-rich cobalt centers. Additional information on the used theoretical methodologies is provided in the Computational Methods section of this paper.

First, the coordination environment of the ^2^[Co^II^(P)]^4–^ (P = either OMeP or F16P) systems was investigated, specifically addressing whether they exhibited a preference for a square planar environment or if axial water ligands could effectively coordinate to the cobalt center. It appears that for both catalysts, the square-planar low-spin ^2^[Co^II^(P)]^4–^ complex was slightly more stable than a five-coordinated, low-spin ^2^[Co^II^(P)(H_2_O)]^4–^ complex by 3.5 and 1.7 kcal mol^–1^ for the F16P and OMeP catalysts, respectively (detailed in ESI, Table S2). We also optimized the high-spin ^4^[Co^II^(OMeP)]^4–^ complex, which was found to be significantly higher in energy than the low-spin analogue, by 16.4 kcal mol^–1^ (Table S2). Optimization of the high-spin ^4^[Co^II^(F16P)]^4–^ complex failed due to convergence issues in the self-consistent field optimization, however, this species is believed to be higher in energy than the low-spin complex. Consequently, for the remainder of the paper, the ^2^[Co^II^(P)]^4–^ complexes, without water ligands are considered as the catalysts resting states.

We then analyzed the coordination environment of these complexes after the second reduction. During the optimization of the 2-electron reduced ^1^[Co^I^(P)(H_2_O)]^5–^ species, the axial water ligand dissociated completely, underlining the preference of the d8 metal complex for a square planar environment. We also considered the triplet analogues of the 2e^–^ reduced species, however, our calculations reveal a preference of over 13 kcal mol^–1^ for the low-spin ^1^[Co^I^(P)]^5–^ complexes over the high-spin ^3^[Co^I^(P)]^5–^ analogues in both systems (see Table S2). This is in line with NMR measurements reported in the literature, where Co(I)-porphyrins exhibited a singlet spin multiplicity.^37^

**Figure 6:**
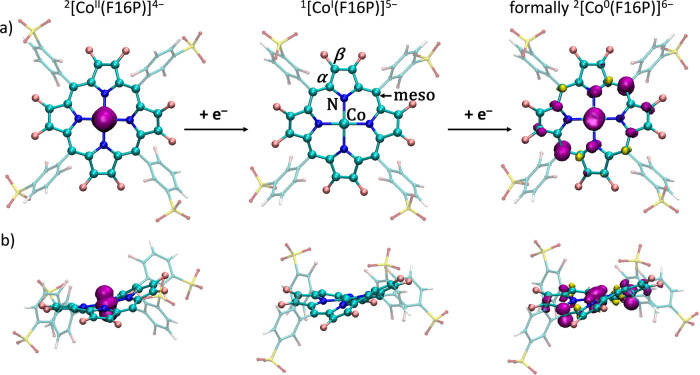
Top (a) and side (b) views of the optimized structures of the [Co(F16P)]^*n*−^ catalyst complex in the Co^II^, Co^I^ and formally Co^0^ oxidation states. For the complexes with unpaired electrons, the spin density is visualized with an isosurface value of 0.003. The ^1^[Co^I^(F16P)]^5–^ intermediate contains atom labels that are used in the text to distinguish the five distinct protonation sites on the cobalt porphyrin complex.

After establishing the most favorable spin states and coordination environment, the electronic structure and geometries of two investigated systems were analyzed in the Co^II^, Co^I^ and formally Co^0^ oxidation states. Figure 6 presents the optimized geometries of the three intermediates of the [Co(F16P)]^3–^ catalyst, while the structures for the [Co(OMeP)]^3–^ catalyst can be found in the Figure S18. Visualization of the spin-density of the species upon a third reduction to what is formally a Co^0^ complex, revealed that the added electron was delocalized over the π-system of the porphyrin ring and the d orbital of the cobalt center (Figure 6, right panel), which can hence be interpreted as an intermediate species between the two localized forms ^2^[Co^I^(P^•–^)]^6–^ and ^2^[Co^0^(P)]^6–^.

As in principle all reduction steps might be coupled, or not, to proton transfer, the p*K*_b_ values were calculated for the two catalysts in the Co^I^ and formally Co^0^ oxidation states, considering five plausible protonation sites. The considered protonation sites were the cobalt center, the nitrogen on the porphyrin ligand, the porphyrin α-position, the β-position as well as the meso-position. These different positions susceptible to protonation are indicated with specific labels in Figure 6, top middle panel. For the p*K*_b_ calculations of the 2e^–^ reduced Co^I^ species, we considered high- and low-spin options, as well as potential coordination of an axial aqua ligand to the complex. The calculated p*K*_b_ values are presented in Table 1, while the relative energies of all considered intermediates are detailed in the ESI, Table S2.

**Table 1:** Calculated p*K*_b_ Values for Protonation on the Metal Center and on the Nitrogen, α-, β- and Meso-Position on the Porphyrin Ligand

	Co	N	α	β	meso
^1^[Co^I^(F16P)]^5–^	15.8	22.9	24.5	20.9	23.9
^2^[Co^0^(F16P)]^6–^	6.7	12.6	3.3	–2.5	3.7
^1^[Co^I^(OMeP)]^5–^	9.7	17.9	20.1	13.7	12.8
^2^[Co^0^(OMeP)]^6–^	2.0	10.9	2.5	–2.9	–5.9

Table 1 shows that the metal center of the ^1^[Co^I^(P)]^5–^ species emerges as the most basic site for both catalysts after the second reduction, with p*K*_b_ values of 15.8 and 9.7 for the ^1^[Co^II^(F16P)]^4–^ and the ^1^[Co^II^(OMeP)]^4–^ systems, respectively. This is interesting, as it indicates that at pH 4.1 conditions, the electron-rich ^1^[Co^II^(OMeP)]^4–^ catalyst will be protonated at the metal center, while the electron-poor analogue will remain unprotonated. The most favorable protonation site in both systems shifts to the porphyrin macrocycle after the third reduction forming the formally ^2^[Co^0^(P)]^6–^ intermediate. At this stage our calculations showed that protonation of the complexes was exothermic at both pH 7.0 and pH 4.1, with the ligand being more basic than the metal center by about ∼8 and ∼9 p*K*_b_ units for the F16P and OMeP systems, respectively. This is qualitatively consistent with other DFT studies on cobalt porphyrin-catalyzed hydrogen evolution.^37^ Our calculations show that the most favorable protonation site for the electron-poor complex ^2^[Co^0^(F16P)]^6–^, is the β-pyrrole position with a value of −2.5 p*K*_b_ units. In contrast, the meso-position appeared to be more basic for the ^2^[Co^0^(OMeP)]^6–^ system, with value of −5.9 p*K*_b_ units. We attribute the shift toward a preference for the meso-position to the nearby methoxy group in the ortho-position of the benzo-4-sulfonic acid substituent. In the optimized structure, the distance between the added hydrogen atom and the oxygen atom of the methoxy group was 2.32 Å, indicating the possible formation of a hydrogen bond to stabilize the protonated state. Visualization of the spin-densities of the ligand protonated intermediates showed that the excess electron was still largely delocalized over both the Co center and the ligand (Figure S19), indicating once more an intermediate species between the two localized forms ^2^[Co^I^(P–H^•–^)]^5–^ and ^2^[Co^0^(P–H)]^5–^. For the remainder of this paper, we will refer to these complexes as formally ^2^[Co^0^(P–H)]^5–^, in which the P–H ligand represents a hydride bound to a cation radical porphyrin ligand P^•+^.

**Figure 7:**
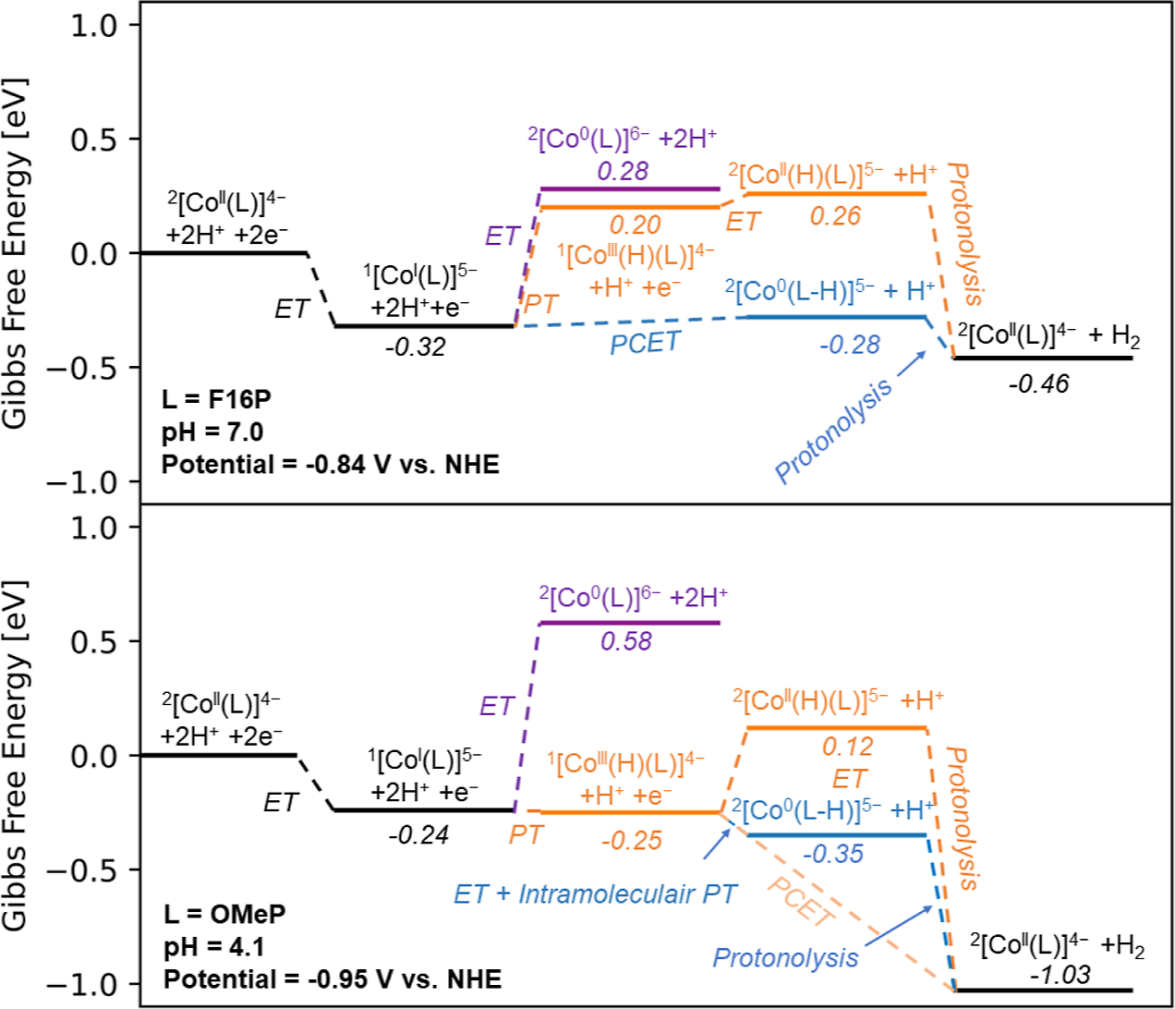
Calculated Gibbs free energy diagrams for the [CoF16P]^3–^ (top) and [CoOMeP]^3–^ (bottom) systems. In the Gibbs free energy diagrams the first reduction step from Co^III^ to Co^II^ is not shown for clarity. [^2^Co^0^(P)]^6–^ notation is formal and in reality, the added charge is delocalized between Co and the Ligand. In the ^2^[Co^I^(P–H)]^5–^ notation, P–H formally corresponds to a cation radical porphyrin ligand P^•+^ and a hydride. The Gibbs free energy diagrams are calculated at the pH for which the catalysts achieved the highest TON, and at the onset potential of the catalytic wave for each catalyst. For the electron-poor [Co(F16P)]^3–^ system this is at pH 7.0 and a potential of −0.84 V vs NHE, while for the [Co(OMeP)]^3–^ system this is at pH 4.1 and a potential of −0.95 vs NHE. Due to the different proton and electron potentials, formation of H_2_ is more exothermic in the bottom panel than for the top panel.

Figure 7 summarizes the most probable mechanistic pathways according to our experimental and theoretical results. It starts from the 1e^–^ reduced, activated catalyst in the Co^II^ oxidation state. The relative Gibbs free energies have been calculated assuming optimal pH values and the experimentally observed catalytic onset potential for both catalysts: pH = 7.0 and a potential of −0.84 V vs NHE for the [Co(F16P)]^3–^ system, and pH = 4.1 and a potential of −0.95 V vs NHE for the [Co(OMeP)]^3–^ system.

Figure 7 shows that after the Co^II^ to Co^I^ reduction, protonation of the electron-poor catalyst ^1^[Co^I^(F16P)]^5–^ to generate a ^1^[Co^III^(H)(F16P)]^4–^ metal hydride is thermodynamically unfavorable by 0.52 eV (orange pathway in top panel). Similarly, the purely electronic reduction to form the formally Co^0^ intermediate is energetically uphill by 0.60 eV (purple pathway). DFT thus predicts the purely electronic reduction of ^1^[Co^I^(F16P)]^5–^ to ^2^[Co^0^(F16P)]^6–^ to lay at −1.44 V vs NHE, in line with CV measurements of the [Co(F8P)]^3–^complex in DMF, which shows an onset at ca. −1.4 V (see Supporting Information, Figure S7a). As both reactions involving only proton or only electron transfer are endergonic, the most favorable pathway involves a concerted process, in which the reduction is coupled to protonation of the porphyrin macrocycle (blue PCET pathway in top panel of Figure 7). We emphasize that the ligand is more basic than the cobalt by 9.2 p*K*_a_ units for the 3-electron reduced ^2^[Co^0^(F16P)]^6–^ complex (see Table 1). Therefore, the proton attaches to the ligand, and not to the cobalt. Subsequently, dihydrogen formation can occur by protonolysis of the hydride ligand, which is exothermic by 0.18 eV.

In contrast, for the electron-rich ^1^[Co^I^(OMeP)]^5–^ catalyst, metal hydride formation following the Co^II^ to Co^I^ reduction is already exothermic, although only by 0.01 eV (orange pathway in bottom panel of Figure 7). DFT thus indicates that for this complex a Co^III^ hydride is formed, after the Co^II^ to Co^I^ reduction step. Subsequently, the purely electronic reduction from this Co^III^ hydride to the Co^II^ hydride is endothermic by 0.37 eV (see Figure 7, orange pathway, formation of ^2^[Co^II^(H)(OMeP)]^5–^). This highly endothermic purely electronic reduction suggests once more that this reduction is coupled to proton transfer. The process becomes exothermic by 0.78 eV once we consider the reduction to be coupled with protonolysis of the metal hydride, yielding H_2_ and regenerating the initial catalyst (Figure 7, orange PCET pathway in bottom panel). Alternatively, the reduction may be coupled with intramolecular proton/hydride transfer from the metal to the porphyrin ligand (Figure 7, blue pathway in bottom panel), which would be exothermic by 0.10 eV.

To verify that H_2_ formation for all hydride intermediates is exothermic, hydricity values for these species were calculated following a thermodynamic cycle proposed in literature (see Supporting Information, Tables S5 and S6).^56,57^ All considered intermediates have hydricity values below 34.2 kcal mol^–1^, indicating that hydrogen formation is thermodynamically feasible.

Overall, p*K*_b_ calculations of the [Co^I^(P)]^5–^ and the formally [Co^0^(P)]^6–^ complexes showed that after formation of Co^I^, the electron-rich ^1^[Co^I^(OMeP)]^5–^ catalyst is protonated at the metal, while the ^1^[Co^I^(F16P)]^5–^ porphyrin is unprotonated. Considering that the experiments were performed in aqueous conditions, it is reasonable to assume that if protonation of the reduced species is thermodynamically downhill, the electron transfer is coupled to proton transfer. Table 2 presents the assignment of the experimental redox couples, based on these assumptions.

**Table 2:** Assignment of the Redox Couples Observed in Experiment

assigned redox couple	calculated redox potential (V vs NHE)	measured redox potential (V vs NHE)^a^
[Co(OMeP)]		
^2^[Co^II^(OMeP)]^4–^ + e– + H^+^ ⇆ ^1^[Co^III^(H)(OMeP)]^4–^	–0.70	–0.78
^1^[Co^III^(H)(OMeP)]^4–^ + e– ⇆ ^2^[Co^II^(H)(OMeP)]^5–^ ⇆ ^2^[Co^II^(OMeP-H_meso_)]^5–^	–1.31 (−0.85)^b^	–0.95
[Co(F16P)]		
^2^[Co^II^(F16P)]^4–^ + e– ⇆ ^1^[Co^I^(F16P)]^5–^	–0.52	–0.53
^1^[Co^I^(F16P)]^5–^ + e– + H^+^ ⇆ ^2^[Co^I^(F16P–H_β_)]^5–^	–0.88	–0.84

a Data according to CV, V vs NHE, measured at pH 4.1 for the [Co(OMe)]^3–^complex and pH 7.0 for the [Co(F16P)]^3–^complex.

b –1.31 V corresponds to the purely electronic reduction to form ^2^[Co^II^(H)(OMeP)]^5–^, without taking proton transfer into account. The number between brackets corresponds to the 1 e^–^reduction coupled to intramolecular proton transfer from the cobalt to the meso-position on the porphyrin ligand.

For the electron-rich ^1^[Co^II^(OMeP)]^4–^ system, the first reversible redox couple is assigned to the proton-coupled reduction of the Co^II^ species to the Co^III^ hydride. The calculated −0.70 V redox potential matches closely the experimentally observed −0.78 V vs NHE. The second catalytic reduction is subsequently attributed to the reduction of the Co^III^ hydride to the Co^II^ hydride. At this stage the calculated potential of −1.31 V is more anodic than the measured potential of −0.95 V. However, we expect that the reduction is coupled to catalytic hydrogen formation via protonolysis of the metal centered hydride. This might lower the onset potential considerably. Alternatively, the reduction could be coupled to intramolecular proton transfer from the metal center to the porphyrin macrocycle, as this site becomes more basic upon reduction of the complex. If this stabilization is considered, the redox potential for the second couple was calculated to lay at −0.85 V, which is very close to the experimentally measured value of −0.95 V.

After the second reduction, the electron-poor complex ^1^[Co^I^(F16P)]^5–^, does not undergo oxidative protonation. Therefore, the current increase at −0.53 V was assigned to the reduction of ^2^[Co^II^(F16P)]^4–^ → ^1^[Co^I^(F16P)]^5–^, and the catalytic wave at −0.84 V is attributed to the proton-coupled electrochemical reduction of ^1^[Co^I^(F16P)]^5–^ → ^2^[Co^0^(F16P–H_β_)]^5–^ and subsequent hydrogen evolution. The calculated potentials of −0.52 and −0.88 V vs NHE very closely match the measured values. In addition, they accurately reproduce the difference between the two onset potentials within an error of 0.05 V.

## 3 Discussion

Our electrochemical study of this series of Co-porphyrin complexes reveals that the electron-richness of the ligand controls the onset potential for the [Co^II^(P)]^4–^ to [Co^I^(P)]^5–^ reduction, after the initial [Co^III^(P)]^3–^ to [Co^II^(P)]^4–^ reduction. However, catalysis does not start at this stage, and a third reduction is needed to lead to catalytic hydrogen evolution. The dependence of this third reduction step on the electronic properties of the porphyrin ring substituents was less straightforward to establish than for the first two reductions. As expected, the electron-richest complex [Co(OMeP)]^3–^ had the most cathodic onset potentials of −1.04 and −0.95 V vs NHE at pH 7.0 and pH 4.1, respectively (Table S1). However, the electron-poorest complex [Co(F16P)]^3–^ showed the least negative onset potential of −0.84 V vs NHE only at pH 7.0. Surprisingly at pH 4.1, it was the electron rich [Co(MeP)]^3–^ that showed the least negative onset potential of −0.74 V vs NHE.

We then proceeded to perform photocatalytic experiments using standard concentrations of 0.1 mM catalyst, 0.5 mM [Ru(bpy)_3_]Cl_2_, 0.1 M ascorbate and TCEP, and light power at 450 nm of 16 mW. Given that the system has a large excess of electron donor (0.1 M) over photosensitizer (0.5 mM), it is reasonable to assume that the photosensitizer is reductively quenched during the photocatalytic process. This is in line with literature, which shows that photocatalytic experiments with ascorbate and TCEP favor reductive quenching of the Ru(bpy)_3_ photosensitizer.^12^ We have experimentally determined the driving force *E*_dr_ value (in V) for electron transfer from the reduced photosensitizer PS^–^ to the Co-porphyrins from the difference between *E*_PS_(PS/PS^–^) and the hydrogen evolution onset potential of the catalyst in electrochemical conditions. We find that all investigated cobalt complexes have a catalytic onset potential that is higher than the *E*_PS_(PS/PS^–^) value. This implies that the reduced photosensitizer can act as electron source for all reductions in the catalytic cycles of the investigated cobalt catalysts.

**Figure 8:**
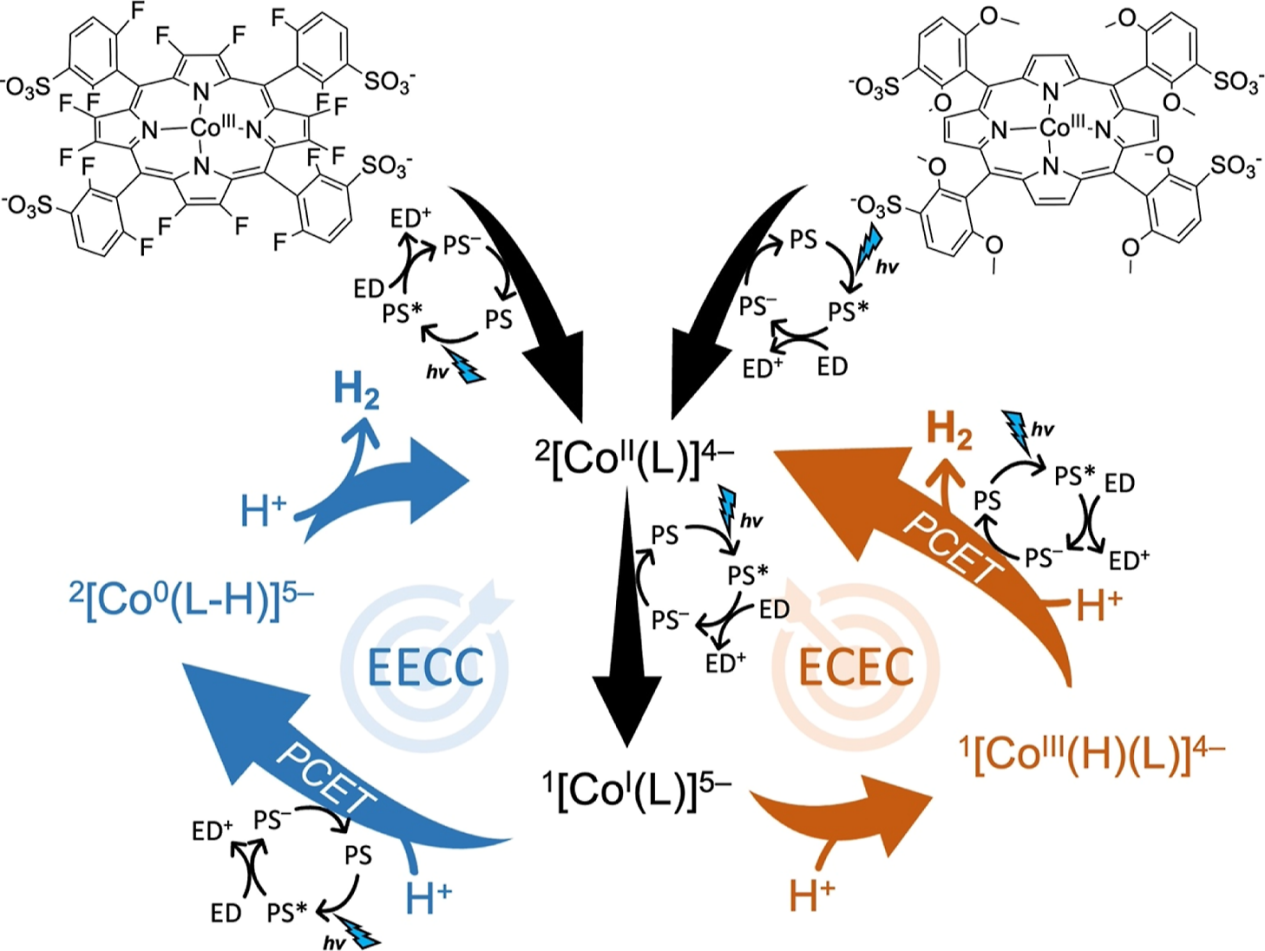
Reaction mechanism for photocatalytic hydrogen evolution starting from [Co^III^(F16P)]^3–^ (left) and [Co^III^(OMe)]^3–^ (right).

The decrease in *E*_dr_ for [Co(F16P)]^3–^ from 420 mV at pH 7.0 to 380 mV at pH 4.1 suggests that electron transfer from the photoreduced species PS^–^ to [Co(F16P)]^3–^ may be faster at pH 7.0 than at pH 4.1. However, our kinetic studies in photocatalytic conditions demonstrated that the rate of the photocatalytic system using [Co(F16P)]^3–^ as HEC at pH 7.0 was limited by the activity of the catalyst rather than the electron transfer between PS^–^ and the HEC. At pH 4.1, using identical concentrations and light intensity, the catalytic step of the photocatalytic system using [Co(OMeP)]^3–^ as HEC was limited by the photon flux, rather than by electron transfer or the activity turnover of the catalyst. Overall, at both pH 7.0 and 4.1, the onset potential of the catalyst in electrocatalytic conditions had very little predictive value in terms of its activity in photocatalytic conditions. The different performances of the photocatalytic systems using [Co(OMeP)]^3–^ at pH 4.1 and of [Co(F16P)]^3–^ at pH 7.0 is primarily attributed to differences in their HER catalytic mechanisms, as is further supported by DFT calculations. A schematic illustration of the proposed mechanisms is provided in Figure 8.

Consistent with CV experiments, our DFT calculations indicated that the first steps in the catalytic cycle for the two catalysts are identical. After an initial Co^III^ to Co^II^ reduction, the low-spin ^2^[Co^II^(P)]^4–^ complexes are reduced to form a low-spin ^1^[Co^I^(P)]^5–^ intermediate. Subsequently, the mechanisms of the two investigated catalysts diverge. The p*K*_b_ value of 9.7 of the [Co^I^(OMeP)]^5–^ metal center suggested that at pH 4.1 the metal center is protonated, thus forming the metal hydride species ^1^[Co^III^(H)(OMeP)]^4–^ (Table 1). At pH 7.0, most complexes remain unprotonated according to our calculations, although we note that p*K*_b_ calculations are very sensitive to the accuracy in the DFT method and the calculated solvation energies, introducing potential errors up to 2–3 p*K*_a_ units.^58,59^ Distinctively, the much higher p*K*_b_ value of 15.8 of the low-spin ^1^[Co^I^(P)]^5–^ intermediate for [Co(F16P)]^5–^ reveals that this complex remains unprotonated in both buffer solutions. This bifurcation in the mechanisms after the second Co^II^ → Co^I^ reduction, explains the observed trend in the third reduction waves in electrochemical measurements. At pH 4.1, the [Co(MeP)]^3–^ catalyst was found to have the lowest catalytic onset potential, followed by [Co(F16P)]^3–^, then [Co(F8P)]^3–^, and finally [Co(OMeP)]^3–^ (see Supporting Information, Table S1). From this, we thus conclude that for the electron-poor [Co(F16P)]^3–^ and [Co(F8P)]^3–^ catalyst, the third reduction corresponds to the ^1^[Co^I^(P)]^5–^ → ^2^[Co^0^(P–H)]^5–^ proton-coupled reduction step (EECC mechanism, Figure 8, left). In contrast, we conclude that for the electron-rich catalysts [Co(MeP)]^3–^ and [Co(OMeP)]^3–^, the proton-coupled reduction of ^1^[Co^III^(H)(P)]^4–^ is observed, which then yields the ^2^[Co^II^(P)]^4–^ complex and H_2_ (ECEC mechanism, Figure 8, right).

The divergence in the mechanisms is further supported by the following arguments. The calculated redox potentials for the second and third reduction steps with the electron poor [Co(F16P)]^3–^ catalyst closely match the measured potentials, validating the correct assignment of these redox couples shown in Table 2. A reduction wave was observed at the foot of the catalytic onset for the electron-poor complexes, which we attribute to a proton-coupled reduction of ^1^[Co^I^(F16P)]^5–^ → ^2^[Co^0^(F16P–H)]^5–^. The observation of this reduction wave at a similar potential in the LSV of both [Co(F16P)]^3–^ and [Zn(F16P)]^4–^ (Figure 3) validates the DFT prediction that both the electron and the proton react with the ligand rather than the metal. Since the Zn metal is redox inactive, this reduction must be ligand-based. Based on the DFT calculations of the cobalt complex, we expect that the [Zn(F16P)]^4–^ complex is also protonated at the ligand, most likely again at the β-pyrrole position. This ligand-based reductive protonation explains the photocatalytic activity of the electron-poor zinc [Zn(F16P)]^4–^ complex, which we would otherwise expect to be redox inactive. CV measurements of the Zn complexes show a catalytic onset around −1.2 V (see Figure 3d), which we thus assign to electron transfer coupled to proton transfer, generating H_2_. Finally, also the CV measurements of the electron poor [Co(F8P)]^3–^ in DMF solution, support the idea that the measured third reduction is strongly coupled to proton transfer in the presence of water. For this complex, the purely electronic reduction from [Co^I^(P)]^5–^ to formally [Co^0^(P)]^6–^ was observed at ca. −1.4 V in pure DMF (Figure S7a), which aligns well with DFT calculations predicting a value of −1.44 V for the purely electronic reduction of [Co^I^(F16P)]^5–^ → [Co^0^(F16P)]^6–^. With increasing water content, the reduction shifted toward more positive potentials, indicating that electron transfer is coupled to proton transfer in water. Our DFT results further demonstrate that the complex with a protonated ligand can act as a hydride source for hydrogen formation (see Supporting Information, Table S5). Although the hydricity of carbons is generally very weak, the system studied here presents an exception. The third reduction of the complex breaks the aromaticity of the porphyrin ring, which is subsequently restored upon hydride donation. This restored aromaticity provides a strong thermodynamic driving force for hydrogen evolution.

On the other hand, our DFT calculations support the idea that the electron-rich [Co(OMe)]^3–^ is protonated after the second reduction to form the Co^III^ hydride species, demonstrated by the p*K*_b_ calculations as well as the calculated redox potential, which again closely matches the assigned redox couple Co^II^ → Co^III^–H. In addition, the quenching of the catalytic activity of the [Zn(OMe)]^4–^ system strengthens our belief that catalytic hydrogen generation with [Co(OMe)]^3–^, occurs via a metal-based pathway, which is a characteristic of many metal-based catalysts.^4,9^

The DFT results reported in this paper qualitatively agree with prior studies by Hammes-Schiffer et al., who showed that electrochemical reduction of a Co(I)-porphyrin complex breaks aromatization in the porphyrin ring, thereby making the ligand more basic than the metal center.^37^ Similarly, Nocera and co-workers have spectroscopically shown that in HER conditions, Ni(II)-porphyrins are readily protonated at the meso-carbon position following two initial reduction steps.^60^ Interestingly, the resulting species does not produce hydrogen, but rather undergoes further hydrogenation. In weakly acidic conditions, the reported complex intermolecularly produced Ni isobacteriochlorin (hydrogenated 4 times on the β-carbon position), while in strongly acidic conditions also higher hydrogenation products were obtained. This is particularly interesting as it gives us a clue as to why the electron-poor [Co(F16P)]^3–^ system loses activity in conditions with pH lower than 6. Even though for the highly anionic intermediate ^2^[Co^II^(F16P–H_β_)]^5–^ any intermolecular electron or proton transfer will probably be difficult, acidic conditions might still trigger further hydrogenation of the macrocycle, lowering the yield for hydrogen evolution.

Overall, the electrochemical, photocatalytic and kinetic experiments presented in this work, in combination with the detailed DFT analysis, show that the synthesized complexes can photocatalytically evolve hydrogen via two distinct mechanisms. The first mechanism is characterized by a metal hydride formation after the Co^II^ → Co^I^ reduction and performs better in acidic conditions, which is typical for proton reduction catalysis. The data provide converging evidence that this mechanism is active for the electron-rich complexes in acidic conditions. In contrast, the second mechanism does not involve a metal hydride, but rather catalyzes the hydrogen evolution via the ligand following a third reduction of the [Co^III^(P)]^3–^ complex. Interestingly, this ligand-based mechanism allows for considerable hydrogen evolution rates at neutral pH conditions with the electron-poor [Co(F16P)]^3–^ catalyst. In more acidic conditions, however, the performance of the electron-poor catalyst was strongly diminished. We tentatively attribute this to the detrimental hydrogenation of the porphyrin macrocycle, which has been extensively reported in previous studies on similar porphyrins complexes bearing Ni.^60^

## 4 Conclusion

A series of water-soluble, tetra-sulfonated Co(III)- and Zn(II)-porphyrin complexes were prepared and tested as catalysts for homogeneous photocatalytic hydrogen evolution from water in neutral and acid conditions. Their catalytic activity and catalytic mechanisms were found to be strongly dependent on the presence of electron-withdrawing or electron-donating substituents on the porphyrin. For the electron-richest catalyst [Co(OMeP)]^3–^, which in theory has a stronger driving force to reduce water than electron-poor complexes, higher photocatalytic activities were observed only at low pH (4.1). In neutral conditions, its activity was much lower and vanished when the cobalt center was replaced by zinc. In contrast, the electron-poorest catalyst [Co(F16P)]^3–^ showed low photocatalytic hydrogen evolution at pH 4.1 but outperformed [Co(OMeP)]^3–^ at neutral pH. Overall, comparable photocatalytic H_2_ production quantum yields were obtained with [Co(OMeP)]^3–^ at pH 4.1 and [Co(OMeP)]^3–^ at pH 7.0. Based on a combination of electrochemical studies, kinetic investigations and extensive DFT modeling, we explain these differences as follows: starting from the ^2^[Co^II^(P)]^4–^ resting state, the first reduction to ^1^[Co^I^(P)]^5–^ is common to both catalyst types. However, in the next step the catalytic mechanisms diverged. In the electron-rich catalyst the metal center was basic enough to be protonated to form a metal hydride intermediate ^1^[Co^III^(H)(P)]^5–^. Subsequently, the catalytic wave observed experimentally was assigned to a proton-coupled reduction of ^1^[Co^III^(H)(P)]^5–^ to recover the ^2^[Co^II^(P)]^4–^ resting state and H_2_, which corresponded overall to a ECEC mechanism. The electron-poor catalyst was much less basic at the metal center than the electron rich catalyst and needed to be reduced by an additional electron before it could accept a proton. This model was consistent with electrochemical experiments showing a second reduction wave at the foot of the catalytic onset. It thus formed a ligand-protonated intermediate formally written ^2^[Co^0^(P–H)]^5–^ where P–H corresponds to a cation radical porphyrin ligand P^•+^ and a hydride. This intermediate in turn can react with a second proton to form H_2_ and recover the resting state in a EECC mechanism. This second mechanism was confirmed by the observation that when using [Zn(F16P)]^3–^ instead of [Co(F16P)]^3–^, part of the photocatalytic activity was retained. Overall, this work represents a significant advance in the understanding of how molecular hydrogen evolution catalyst should be designed. Although for catalytic H_2_ evolution systems at low pH it is better, as usually acknowledged, to use electron-rich catalysts, for neutral conditions (pH 7.0) it is in fact better to have electron-poor cobalt H_2_ evolution catalysts. In water splitting systems coupling photocatalytic HEC and oxygen evolution catalyst near pH 7.0, electron-poor proton reduction catalysts may represent a better optimum than the usually proposed electron-rich catalysts.

## 5 Materials and Methods

All reagents were purchased from Sigma-Aldrich and used as received unless otherwise noted. ^1^H NMR spectra were recorded with a Bruker 400DPX-liq spectrometer operating at 400 MHz. ^19^F NMR spectra were recorded with a Bruker 500DPX spectrometer operating at 500 MHz. High-resolution mass spectrometric measurements were performed with a Bruker Fourier Transform Ion Cyclotron Resonance Mass Spectrometer APEX IV installed at Leiden University. Elemental analyses were performed at the Mikroanalytisches Laboratorium Kolbe, Germany. Electronic absorption spectra were obtained with a Varian Cary 60 spectrophotometer, with samples kept at 25 °C during the spectrophotometric measurements. The LED optical power was measured using an OPHIR Nova-display laser power meter.

### 5.1 Synthesis

#### 5.1.1 Trisodium 5,10,15,20-Tetrakis(2,6-dimethoxyl-3-sulfonatophenyl)porphyrin cobalt(III) (Na_3_[Co(OMeP)])

The ligand Na_4_[H_2_(OMeP)] (115 mg, 0.09 mmol) and Milli-Q water (40 mL) were placed under N_2_ in a 100 mL round-bottom flask equipped with a magnetic stirring bar and a condenser. A solution of cobalt(II) sulfate heptahydrate (126 mg, 0.45 mmol) in Milli-Q water (10 mL) was added to the stirred solution, and the mixture was refluxed for 12 h under N_2_. After cooling to room temperature, water was rotary evaporated at 40 °C and the residue was redissolved in cold methanol (15 mL). After filtration on a filter paper, methanol was rotary-evaporated, the residue was redissolved in cold methanol (5 mL). The solution was filtered by filter paper, rotary-evaporated, and the crude product was redissolved in Milli-Q water (5 mL) then passed onto an Amberlite IR 120 Na^+^ form ion-exchange resin column (10 cm), washed with Milli-Q water and further purified on Sephadex-20H size exclusion chromatography to remove excess cobalt sulfate (methanol). Methanol was finally rotary evaporated and the solid dried in vacuo. Yield (115 mg, 88%); ^1^H NMR (400 MHz, CD_3_OD): δ 9.37–9.19 (m, 8H; β-pyrrole-H), 8.48–8.40 (m, 4H; *p*-Ph-H), 7.52–7.15 (m, 4H; *m*-Ph-H), 3.92–3.49 (m, 12 H; OCH_3_), 3.24–2.29 ppm (m, 12 H; OCH_3_); HRMS (ESI): *m*/*z* calcd for C_52_H_44_N_4_CoO_20_S_4_^+^: 1231.0758 [M – 3Na + 4H]^+^; found: 1231.0750; elemental analysis calcd (%) for C_52_H_40_N_4_Na_3_CoO_20_S_4_·Na_2_SO_4_·H_2_O: C 42.86, H 2.91, N 3.85; Found: C 43.01, H 2.79, N 3.84. UV–vis (H_2_O): λ_max_(ε in M^–1^ cm^–1^) 426 nm (8.8 × 10^4^), 542 nm (5.8 × 10^3^).

#### 5.1.2 Trisodium 5,10,15,20-Tetrakis(2,6-dimethyl-3-sulfonatophenyl)porphyrin cobalt(III) (Na_3_[Co(MeP)])

Tetrasodium 5,10,15,20-tetrakis(2,6-dimethyl-3-sulfonatophenyl)-21*H*,23*H*-porphyrin·9H_2_O (130 mg, 0.10 mmol) and Milli-Q water (40 mL) were placed under N_2_ in a 100 mL round-bottom flask equipped with a magnetic stirring bar and a condenser. A solution of cobalt(II) sulfate heptahydrate (141 mg, 0.50 mmol) in Milli-Q water (10 mL) was added to the stirred solution and the mixture was refluxed for 12 h under N_2_. After cooling to room temperature, water was rotary evaporated, and the residue was redissolved in cold methanol (15 mL). After filtration on a filter paper, methanol was rotary-evaporated, the residue was redissolved in cold methanol (5 mL), the solution was filtered a second time by filter paper and rotary-evaporated, then the crude product was then passed onto an Amberlite IR 120 Na^+^ form ion-exchange resin column (10 cm), washed with Milli-Q water and finally purified on Sephadex-20H size exclusion chromatography (methanol). Methanol was finally rotary evaporated and the solid dried in vacuo. Yield (109 mg, 90%); ^1^H NMR (400 MHz, CD_3_OD): δ 9.18–9.09 (m, 8H; β-pyrrole-H), 8.43–8.38 (m, 4H; *p*-Ph-H), 7.68–7.55 (m, 4H; *m*-Ph-H), 2.42–2.09 (m, 12H; CH_3_), 2.07–1.74 ppm (m, 12H; CH_3_); HRMS (ESI): *m*/*z* calcd for C_52_H_43_N_4_NaCoO12S4^+^: 1125.0985 [M – 2Na + 3H]^+^; found: 1125.0977; elemental analysis calcd (%) for C_52_H_40_N_4_Na_3_CoO_12_S_4_·2H_2_O: C 51.83, H 3.68, N 4.65; found: C 52.08, H 3.43, N 4.66. UV–vis (H_2_O): λ_max_(ε in M^–1^ cm^–1^) 428 nm (2.3 × 10^5^), 545 nm (1.2 × 10^4^).

#### 5.1.3 Trisodium 2,3,7,8,12,13,17,18-Octafluoro-5,10,15–20-tetrakis(2,6-difluoro-3-sulfonato-phenyl)porphyrin cobalt(III) (Na_3_[Co(F16P)])

Tetrasodium 2,3,7,8,12,13,17,18-octafluoro-5,10,15,20-tetrakis(2,6-difluoro-3-sulfonatophenyl)-21*H*,23*H*-porphyrin·5H_2_O (140 mg, 0.10 mmol) and Milli-Q water (40 mL) were placed under N_2_ in a 100 mL round-bottom flask equipped with a magnetic stirring bar and a condenser. A solution of cobalt(II) sulfate heptahydrate (141 mg, 0.50 mmol) in Milli-Q water (10 mL) was added to the stirred solution and the mixture was refluxed for 12 h under N_2_. After cooling to room temperature, the water was rotary evaporated, and the residue was redissolved in cold methanol (15 mL). After filtration over a filter paper, methanol was rotary-evaporated, the residue was redissolved in cold methanol (5 mL), and filtered by filter paper again. The filtrate was rotary-evaporated and the [Co(F16P)]^3–^ complex was then passed through an Amberlite IR 120 Na^+^ form ion-exchange resin column (10 cm) and washed with Milli-Q water before being purified on Sephadex-20H size exclusion chromatography (eluent: methanol). Methanol was finally rotary evaporated and the solid dried in vacuo. Yield (135 mg, 90%); ^1^H NMR (400 MHz, CD_3_OD): δ 6.80 (s, 4H; *p*-Ph-H), 5.60–5.37 ppm (m, 4H; *m*-Ph-H); ^19^F NMR (471 MHz, CD_3_OD): δ −111.97 (d, *J* = 236.0 Hz, 8F; β-pyrrole-F), −112.74 (s, 4F; *o*-F), −113.26 ppm (s, 4F; *o*-F); HRMS (ESI): *m*/*z* calcd for C_44_H_12_F_16_N_4_CoO_12_S_4_^+^: 1278.8406 [M – 3Na + 4H]^+^; found: 1278.8407; elemental analysis calcd (%) for C_44_H_8_F_16_N_4_Na_3_CoO_12_S_4_·Na_2_SO_4_·H_2_O: C 35.12, H 0.67, N 3.72; found: C 35.06, H 0.61, N 3.82. UV–vis (H_2_O): λ_max_(ε in M^–1^ cm^–1^) 402 nm (1.4 × 10^5^), 535 nm (6.3 × 10^3^).

#### 5.1.4 Tetrasodium 5,10,15,20-Tetrakis(2,6-dimethoxyl-3-sulfonatophenyl)porphyrin zincate(II) (Na_4_[Zn(OMeP)])

The ligand Na_4_[H_2_(OMeP)] (115 mg, 0.09 mmol) and Milli-Q water (40 mL) were placed under N_2_ in a 100 mL round-bottom flask equipped with a magnetic stirring bar and a condenser. A solution of zinc(II) chloride (61 mg, 0.45 mmol) in Milli-Q water (10 mL) was added to the stirred solution, and the mixture was refluxed for 12 h under N_2_. After cooling to room temperature, water was rotary evaporated at 40 °C and the residue was redissolved in cold methanol (15 mL). After filtration on a filter paper, methanol was rotary-evaporated, the residue was redissolved in cold methanol (5 mL). The solution was filtered by filter paper, rotary-evaporated, and the crude product was redissolved in Milli-Q water (5 mL) then passed onto an Amberlite IR 120 Na^+^ form ion-exchange resin column (10 cm), washed with Milli-Q water and further purified on Sephadex-20H size exclusion chromatography to remove excess zinc chloride (methanol). Methanol was finally rotary evaporated and the solid dried in vacuo. Yield (107 mg, 85%); ^1^H NMR (400 MHz, CD_3_OD): δ 8.82–8.77 (m, 8H; β-pyrrole-H), 8.40–8.35 (m, 4H; *p*-Ph-H), 7.31–7.23 (m, 4H; *m*-Ph-H), 3.67–3.54 (m, 12 H; OCH_3_), 3.07–2.76 ppm (m, 12 H; OCH_3_); HRMS (ESI): *m*/*z* calcd for C_52_H_45_N_4_ZnO_20_S_4_^+^: 1237.0796 [M – 4Na + 5H]^+^; found: 1237.0792; elemental analysis calcd (%) for C_52_H_40_N_4_Na_4_ZnO_20_S_4_·4H_2_O: C 44.66, H 3.46, N 4.01; found: C 44.79, H 3.19, N 3.99. UV–vis (H_2_O): λ_max_(ε in M^–1^ cm^–1^) 422 nm (1.4 × 10^5^), 557 nm (6.5 × 10^3^).

#### 5.1.5 Tetrasodium 5,10,15,20-Tetrakis(2,6-dimethyl-3-sulfonatophenyl)porphyrin zincate(II) (Na_4_[Zn(MeP)])

Tetrasodium 5,10,15,20-tetrakis(2,6-dimethyl-3-sulfonatophenyl)-21*H*,23*H*-porphyrin·9H_2_O (130 mg, 0.10 mmol) and Milli-Q water (40 mL) were placed under N_2_ in a 100 mL round-bottom flask equipped with a magnetic stirring bar and a condenser. A solution of zinc(II) chloride (68 mg, 0.50 mmol) in Milli-Q water (10 mL) was added to the stirred solution and the mixture was refluxed for 12 h under N_2_. After cooling to room temperature, water was rotary evaporated, and the residue was redissolved in cold methanol (15 mL). After filtration on a filter paper, methanol was rotary-evaporated, the residue was redissolved in cold methanol (5 mL), the solution was filtered a second time by filter paper and rotary-evaporated, then the crude product was then passed onto an Amberlite IR 120 Na^+^ form ion-exchange resin column (10 cm), washed with Milli-Q water and finally purified on Sephadex-20H size exclusion chromatography (methanol). Methanol was finally rotary evaporated and the solid dried in vacuo. Yield (110 mg, 90%); ^1^H NMR (400 MHz, CD_3_OD): δ = 8.63–8.59 (m, 8H; β-pyrrole-H), 8.35 (m, 4H; *p*-Ph-H), 7.56–7.49 (m, 4H; *m*-Ph-H), 2.40–2.26 (m, 12H; CH_3_), 1.94–1.79 ppm (m, 12H; CH_3_); HRMS (ESI): *m*/*z* calcd for C_52_H_45_N_4_ZnO_12_S_4_^+^: 1109.1203 [M – 4Na + 5H]^+^; found: 1109.1204; elemental analysis calcd (%) for C_52_H_40_N_4_Na_4_ZnO_12_S_4_·H_2_O: C 51.34, H 3.48, N 4.61; found: C 51.54, H 3.39, N 4.61. UV–vis (H_2_O): λ_max_(ε in M^–1^ cm^–1^) 423 nm (6.0 × 10^5^), 557 nm (1.8 × 10^4^), 599 nm (4.6 × 10^3^).

### 5.2 Cyclic Voltammetry, Differential Pulse Voltammetry and Linear Sweep Voltammetry

Cyclic voltammetry (CV), differential pulse voltammetry (DPV) and linear sweep voltammetry (LSV) measurements were performed using an Autolab PGstart10 potentiostat controlled by GPES4 software. CV, DPV and LSV measurements were recorded in 0.1 M sodium phosphate buffer (pH 7.0), 1.0 M sodium phosphate buffer (pH 4.1) or 0.1 M tetrabutylammonium hexafluorophosphate (TBAPF6) DMF solution, using a three-compartment cell with a 0.07 cm2 glassy-carbon electrode as the working electrode, Pt wire as the auxiliary electrode, Ag/AgCl (saturated KCl aq.) as the reference electrode for aqueous solution and saturated calomel electrode (SCE) (saturated KCl aq.) as the reference electrode for DMF solution. To ensure consistency, we followed a rigorous preparation protocol for the glassy-carbon electrode before each test: First, the electrode was polished sequentially with 1.0, 0.3, and 0.05 μm alumina for a total of 6 min. After polishing, the electrode tip was ultrasonicated in distilled water and ethanol for 5 min each. It was then allowed to dry at room temperature in ambient air. To verify cleanliness, we performed cyclic voltammetry (CV) tests in 0.5 M H_2_SO_4_ using a potential range of −1.0 to 1.0 V versus Ag/AgCl, at a scan rate of 50 mV/s under an argon atmosphere. If the CV curves remain stable for 20 consecutive cycles, we consider the electrode surface free of impurities. Otherwise, the polishing process was repeated. Finally, we tested the electrode in a 4 mM ferrocyanide solution. The electrode was deemed suitable for use only if the reversible peak separation was less than 80 mV; otherwise, the polishing and testing procedures were repeated. K_3_[Fe(CN)_6_] was added at the end of the measurements in aqueous solutions as internal standard (E([Fe(CN)_6_]^3–^/[Fe(CN)_6_]^4–^) = +0.361 V vs NHE).^61^ Potential vs SCE is converted +0.241 V vs NHE at 298 K. Unless otherwise indicated, the potential was converted relative to NHE, the solutions were bubbled with high-purity argon for at least 30 min before analysis.

### 5.3 Photoinduced Hydrogen Evolution

Photoinduced hydrogen evolution from water was analyzed by a hydrogen electrode (Unisense H2-NP) controlled by x-5 UniAmp using Logger software. The irradiation source was an OSRAM Opto Semiconductors LD W5SM LED (λ_irr_ = 450 nm, Δλ_1/2_ = 25 nm) with water cooling. All the photochemical hydrogen evolution measurements were carried out in a thermostated (298 K) photochemical reactor (total volume 25.0 mL). [Ru(bpy)_3_]Cl_2_·6H_2_O (1.3 mg, 0.5 mM), the catalyst Na_3_[Co(OMeP)], Na_3_[Co(MeP)], Na_3_[Co(F8P)], Na_3_[Co(F16)], Na_4_[Zn(OMeP)], Na_4_[Zn(MeP)], Na_4_[Zn(F8P)], and Na_4_[Zn(F16P)] (0.1 mM), and ascorbic acid (53 mg, 0.1 M) and tris(2-carboxyethyl)phosphine hydrochloride (86 mg, 0.1 M) were added as solids in the reactor, and dissolved using sodium phosphate buffer (0.1 M, pH 7.0, 3.0 mL), the pH value of the mixture solution was then controlled with sodium hydroxide solid by checking pH. Under constant stirring, the reactor was equipped with 1 rubber septum and 2 silicon septa in order to make an airtight system. The hydrogen electrode was then inserted through the septum, to measure the hydrogen concentration in the head space (gas phase) of the photochemical reactor, and the whole system was deaerated by high-purity argon for at least 30 min. After removing the argon, the hydrogen electrode was calibrated by a four-time injection of 100 μL (4.46 μmol at 1 atm) of high-purity H_2_ into the closed system, thereby limiting the overpressure to <5%; the calibration was adapted with the pressure change using Logger software, affording direct reading of the volume of dioxygen (μL) produced in the gas phase of the reactor (*V*_gas_ = 22.0 mL). Following calibration, the three used septa were replaced by new ones and the hydrogen electrode was reinserted into the system. The system was degassed for 30 min with argon, then data recording was started, first keeping the system in the dark for another 30 min prior to starting light irradiation. Unless otherwise indicated, the data recording was stopped after 39.5 h of light irradiation.

Turnover number and turnover frequency determination. The turnover number (TON) of oxygen evolution was determined by a hydrogen electrode (Unisense OX-NP) controlled by x-5 UniAmp using Logger software. The amount of oxygen formed during 39.5 h illumination was used to calculate the TON. The TON were calculated from the oxygen production data by the following equation
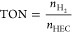
in which *n*_H_2__ is the number of mol of dihydrogen calculated from the volume of the H_2_ produced in the photocatalytic experiment as indicated by the calibrated hydrogen electrode in the gas phase (μL), divided by 22.4 L/mol, and *n*_HEC_ is the number of mol of cobalt or zinc porphyrin catalyst used in the photocatalytic experiment. The TON errors were calculated according to triplicate measurements.

The maximum H_2_ evolution rate (in h^–1^) or the maximum turnover frequency TOF_max_ (in h^–1^) of photocatalytic hydrogen evolution was obtained using Origin 9.1 software by (1) nonlinear curve fitting of the time evolution of the TON, starting at *t* = 30 min for photocatalytic reactions (category: growth/sigmoidal, function: logistic fit); (2) calculating the first derivative TOF = *f*(*t*) using mathematics, differentiate, and (3) identify the maximum value of the H_2_ evolution rate TOF = *f*(*t*), noted TOF_max_ (example of [Co(OMeP)]^3–^ shown in Figure S20). The maximum H_2_ evolution rate and TOF_max_ errors were calculated according to triplicate measurements.

### 5.4 Calculation of Photochemical H_2_ Production Quantum Yield

The H_2_ generation quantum yield was calculated using the TOF_max_, *n*_HEC_ and the rate of photons absorption
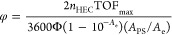
in which TOF_max_ (h^–1^) is the maximum turnover frequency of the photocatalytic hydrogen evolution reaction, *n*_HEC_ (in μmol) is the number of mol of cobalt catalyst used in the photocatalytic experiment, Φ is the photon flux (μmol s^–1^) determined by standard ferrioxalate actinometry (typically 1.05 μmol s^–1^ when using a 16 mW light power),^62^*A*_e_ is the total absorption of the photocatalytic solution at 450 nm (in all experiments *A*_e_ > 3 and the photon absorption probability was ∼1), *A*_PS_ is the absorption of photosensitizer only in buffer at 450 nm (), and 3600 is the number of seconds per hour. In this calculation we assumed 2 photons were needed for each molecule of H_2_ produced. The quantum yield errors were calculated according to triplicate measurements.

### 5.5 Computational Details

Structure optimizations and Hessian calculations were performed within the AMS2021.101 package by SCM.^63^ All structures were optimized using the OPBE density functional approximation,^64^ in an all-electron double-ζ polarized (DZP) Slater-type basis set, including scalar relativistic effects by means of the zero-order regular approximation (ZORA).^65^ Grimme’s third generation dispersion corrections (D3) were used with the Becke-Johnson damping function.^66−68^ Geometries were confirmed to be local minima on the potential energy surface by a Hessian calculation without imaginary frequencies. This vibrational analysis was also used to estimate the thermodynamic corrections in the calculated free energies. The partition function contribution of low frequency vibrations has been modified as described in ref (69). Because AMS2021.101 does not provide the absolute energies required for the p*K*_a_ calculations, Gaussian16.2 single-point energy calculations were performed on the obtained structures.^70^ These calculations were performed using the PBE functional with D3(BJ) dispersion corrections in the def2TZVP basis set.^71−73^ The Gibbs free energy of solvation was calculated with the SMD model and SCF convergence was set to “tight”.^74^ To verify the consistency between the Gaussian16 and AMS results, the energies of the obtained geometries were also re-evaluated by performing a single point calculation in AMS2021, employing the OPBE-D3(BJ) functional in a ZORA triple-ζ Slater-type basis with two polarization functions (ZORA-TZ2P). At this point, the solvation of the complexes in water was accounted for by the implicit conductor like screening model (COSMO).^75−77^ For all calculations the integration quality was improved over the default settings by putting the numerical quality to “good”. It was found that the change in program and density functional affected the one electron reduction potentials of the [^2^Co^II^(F16P)]^4–^ and [^2^Co^II^(OMeP)]^4–^ complexes by less than 0.02 V vs NHE (Table S3).

To assess the functional dependence of the calculated results, the [^2^Co^II^(F16P)]^4–^ to [^1^Co^I^(F16P)]^5–^ reduction potential was re-evaluated with a series of different functionals (see Table S4). These reduction potentials were calculated by performing a single point calculation within the AMS2021.101 program with the functional in question, including D3(BJ) dispersion corrections, in a ZORA-TZ2P basis with COSMO solvation on the OPBE-D3(BJ)/ZORA-DZP structures. Interestingly, all hybrid functionals predict a potential that is significantly more anodic than the experimentally observed potential. This was observed also in earlier DFT studies of cobalt porphyrins.^78,79^ Recent studies have addressed this issue by systematically shifting the calculated reduction potentials to match the experimentally observed potential.^80^ In this work, instead we opted for using a GGA functional, as these functionals have also been shown to correctly predict the singlet Co(I)-porphyrin complexes to be more stable than the triplet analogues.^37^

### 5.6 Thermodynamic Calculations

The Gibbs free energies for all structures in solution were calculated following a Born-Haber thermodynamic cycle
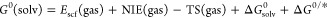
where *G*^0^(solv) is the Gibbs free energy of the solvated structure, *E*_scf_(gas) is the electronic energy from the single point calculation, NIE and TS are the nuclear internal energy and entropy corrections as provided by the vibrational analysis in the gas phase, and Δ*G*_solv_^0^ is the Gibbs free energy of solvation, given by the solvent model. We note that the NIE term includes both the zero-point energy contribution, as well as rotational and translational contributions. The Δ*G*^0/*^ term is introduced to convert the Gibbs free energy in the gas-phase at a pressure of 1 atm to the Gibbs free energy of the solvated molecule at the desired concentration. The term is calculated as
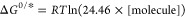
where *R* is the universal gas constant, *T* is temperature (in K) and [molecule] is the concentration of the molecule.^81^ The Gibbs free energies associated with redox reactions were calculated using the following equation
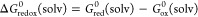
where *G*_red_^0^ and *G*_ox_^0^ were the Gibbs free energies of the relaxed oxidized and reduced species, including protonation steps and ligand dissociations. Subsequently, redox potentials could be calculated directly as
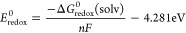
where *n* is the number of transferring electrons and *F* is the Faraday constant. The 4.281 eV term is introduced to translate the absolute potentials to potentials against NHE.^81^ p*K*_a_ values were calculated directly from the Gibbs free energy associated with the dissociation reaction HA → H^+^ + A^–^
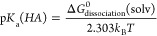
where the (translational) free energy of a proton was taken as −6.23 kcal mol^–1^ and the solvation energy of a proton in water was taken as −264.0 kcal mol^–1^ at 1 atm and 298.15 K standard state conditions.^82,83^*k*_B_ is the Boltzmann constant. The Δ*G*^0/*^ term to convert the Gibbs free energy of the proton in the gas-phase to the Gibbs free energy of a proton in solution at 1 mol L^–1^ is −1.89 kcal mol^–1^.^84^ Finally, the Gibbs free energies associated with the protonation of a metal complex at a specific pH value was calculated as
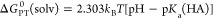
where Δ*G*_PT_^0^(solv) is the Gibbs free energy of the proton transfer reaction in solution.

## Supporting Information Available

NMR spectra, part of cyclic voltammetry, linear sweep voltammetry and photocatalytic hydrogen evolution experiments, photostability and kinetic study, computational details, calculation of maximum H_2_ evolution rate and TOF calculations (PDF)
