# Supplementary material for: Electron-Withdrawing Effects in Cobalt Porphyrin Catalysts Boost Homogeneous Photocatalytic Hydrogen Evolution in Neutral Aqueous Solutions
Source: ACS Catal. 2025 Mar 5;15(6):4681–97. doi: 10.1021/acscatal.5c00788 (PMC11934257; doi:10.1021/acscatal.5c00788)
Supplement: Supplementary file 1 — cs5c00788_si_001.pdf [file cs5c00788_si_001.pdf]

Supplementary Information

Electron-Withdrawing Effects of Cobalt Porphyrin Catalysts Boost Homogeneous Photocatalytic Hydrogen Evolution in Neutral Aqueous Solutions

Authors: Chengyu Liu‡, Titus de Haas‡, Francesco Buda*, and Sylvestre Bonnet*

‡These authors contributed equally to this work.

Affiliation: Leiden Institute of Chemistry, Leiden University, Einsteinweg 55, PO Box 9502, 2333CC Leiden, The Netherlands.

***Corresponding Authors, Email:**

f.buda@lic.leidenuniv.nl (F.B.)

bonnet@chem.leidenuniv.nl (S.B.)

Table of Contents

^{1}H-NMR and ^{19}F-NMR (Figure S1-S6) 2

Cyclic Voltammetry and Linear Sweep Voltammetry (Table S1, Figure S7-S9) 4

Photocatalytic Hydrogen Evolution (Figure S10-S13) 7

Photostability (Figure S14-S16) 9

Kinetic Study (Figure S17) 11

Density Functional Theory (Table S2-S6, Figure S18-S19) 12

Calculation of Maximum H_2 Evolution Rate and Maximum TOF (Figure S20) 17

^{1}H-NMR and ^{19}F-NMR

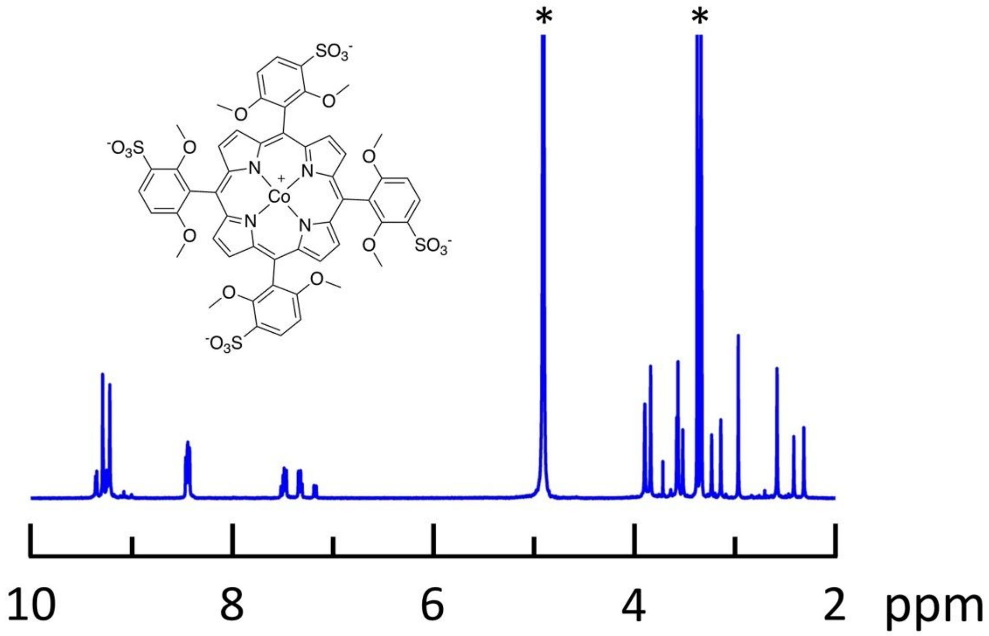

Figure S1 *^{1}H NMR of $[Co(OMeP)]^{3-}$ in MeOD. * Methanol and water.*

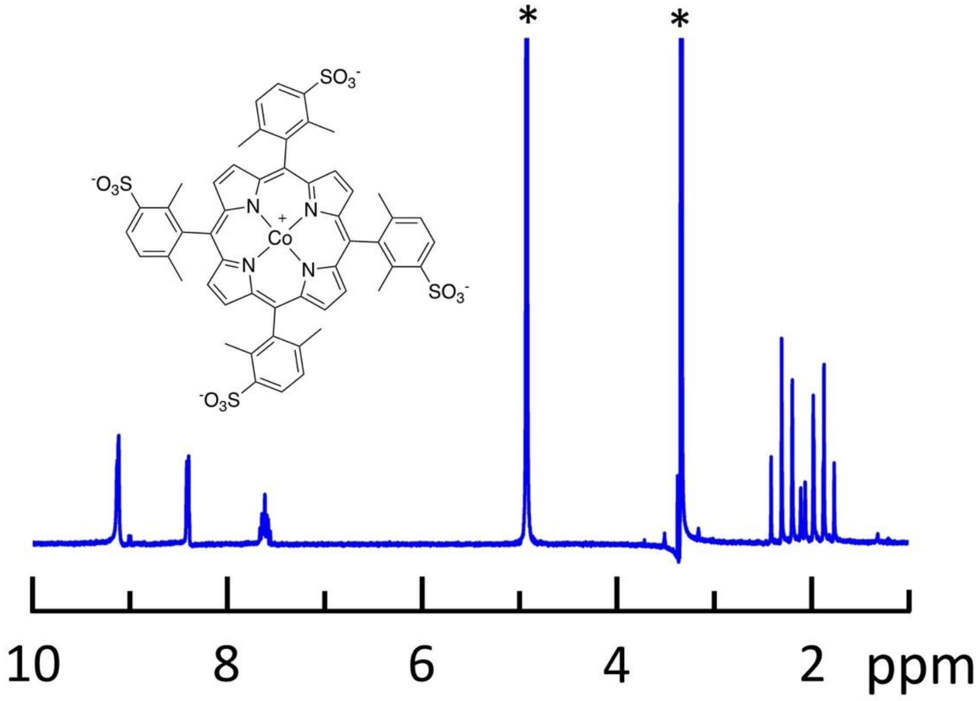

Figure S2 *^{1}H NMR of $[Co(MeP)]^{3-}$ in MeOD. * Methanol and water.*

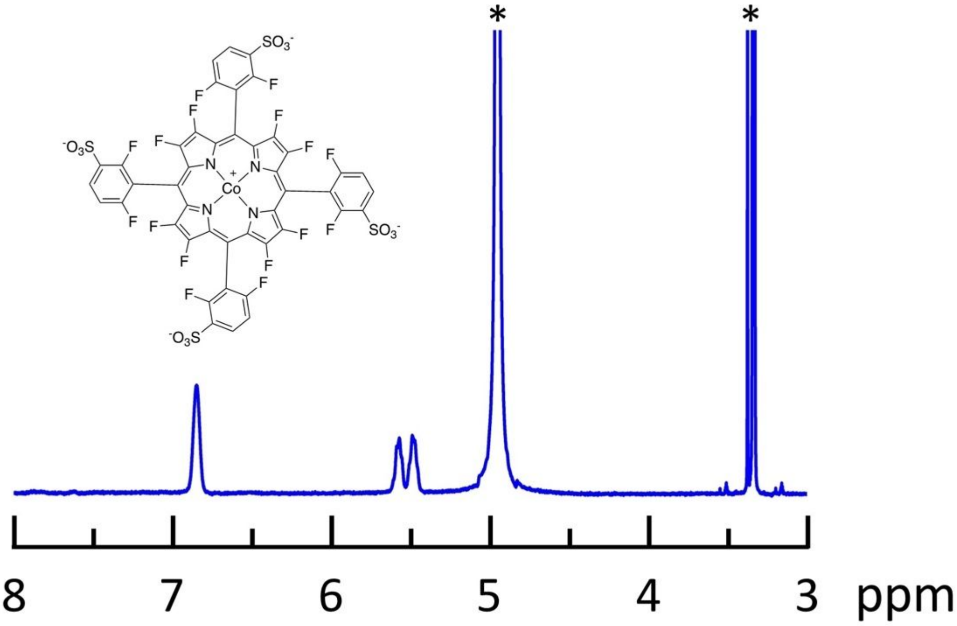

Figure S3 *^{1}H NMR of [Co(F16P)]$^{3-}$ in MeOD. * Methanol and water.*

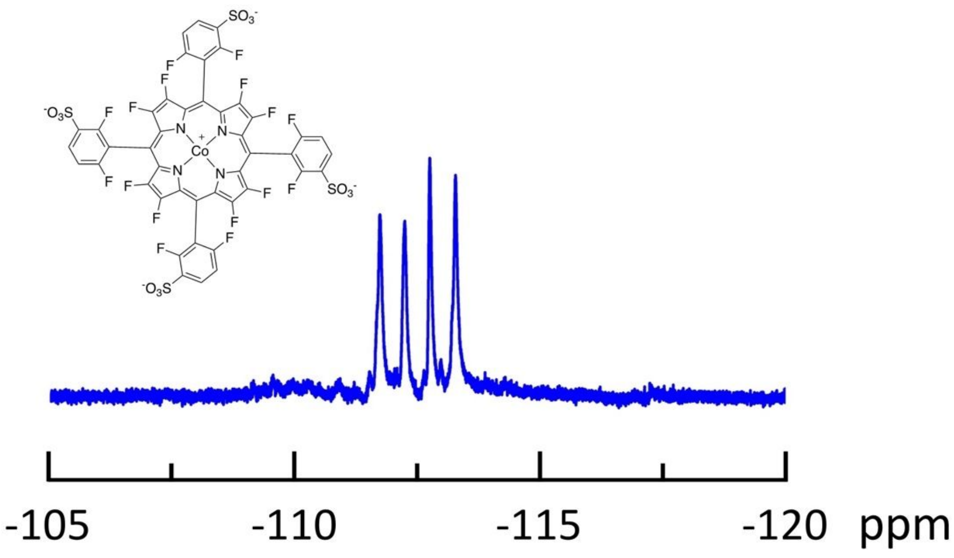

Figure S4 *^{19}F NMR of [Co(F16P)]$^{3-}$ in MeOD.*

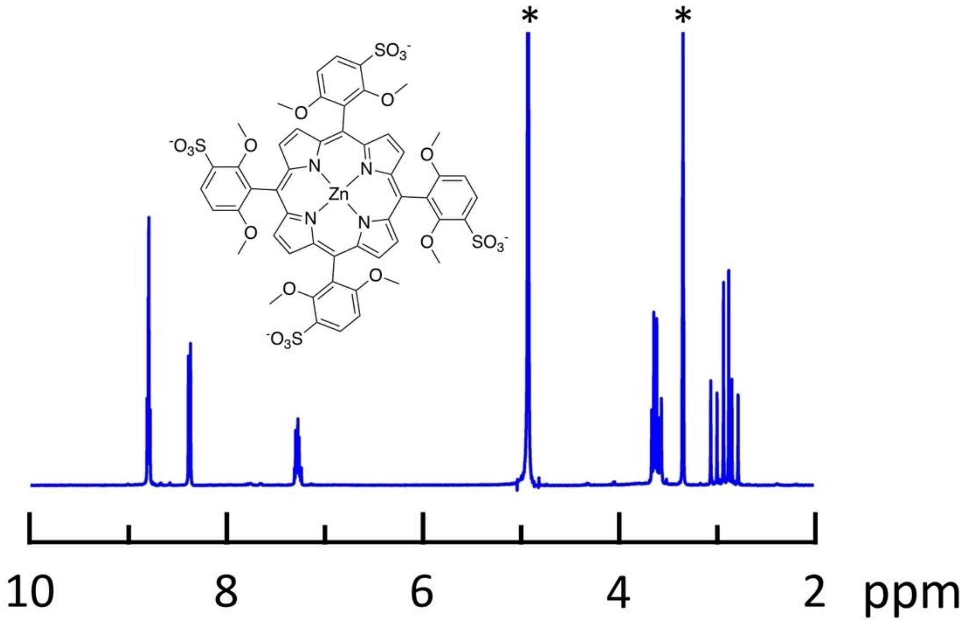

Figure S5 *^{1}H NMR of [Zn(OMeP)]$^{4-}$ in MeOD. * Methanol and water.*

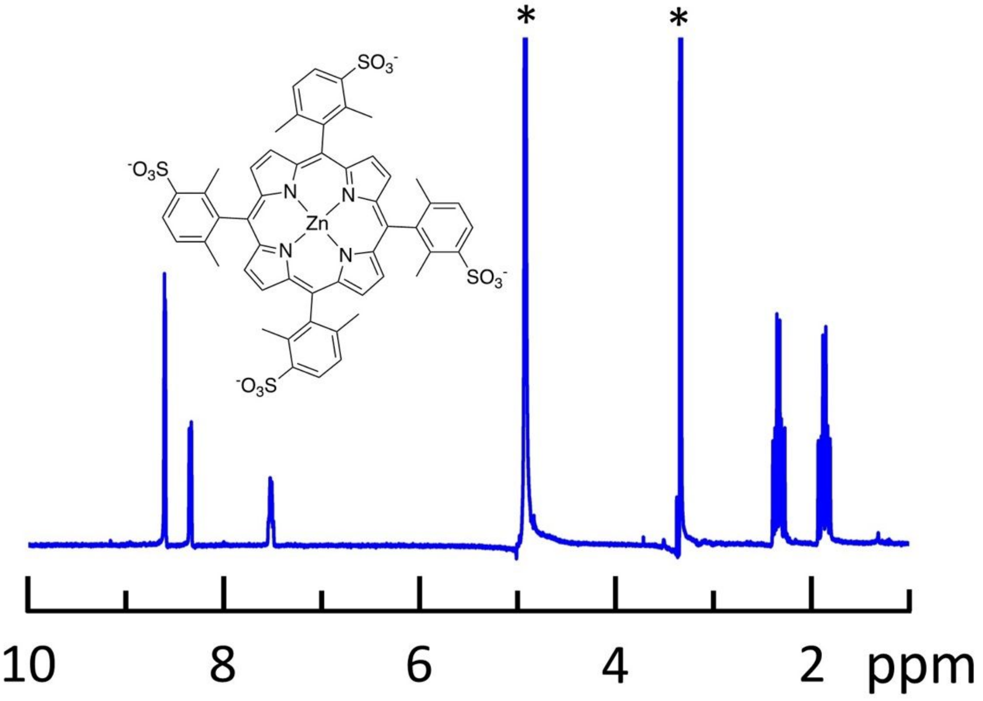

Figure S6 *^{1}H NMR of [Zn(MeP)]$^{4-}$ in MeOD. * Methanol and water.*

Cyclic Voltammetry and Linear Sweep Voltammetry

Table S1 Electrochemical properties of Co-porphyrin complexes at pH 7.0 and pH 4.1.

	**2nd reduction potential at pH 7.0 (at pH 4.1)[a]**	**HER on-set potential at pH 7.0 (at pH 4.1)[b]**	**η at pH 7.0 (at pH 4.1)[c]**	**E_{dr} at pH 7.0 (at pH 4.1)[d]**
**[Co(OMeP)]$^{3-}$**	−0.75 (−0.78)	−1.04 (−0.95)	630 (710)	220 (310)
**[Co(MeP)]$^{3-}$**	−0.70 (−0.73)	−0.95 (−0.74)	540 (500)	310 (520)
**[Co(F8P)]$^{3-}$**	−0.64 (−0.66)	−1.01 (−0.94)	600 (700)	250 (320)
**[Co(F16P)]$^{3-}$**	−0.53 (−0.55)	−0.84 (−0.88)	430 (640)	420 (380)

[a] data according to DPV, V vs. NHE. [b] data according to CV, V vs. NHE. [c] calculated according to the HER on-set potential, in mV. [d] calculated according to $E_{PS}(PS/PS^{-}) = -1.26$ V vs. NHE, in mV.

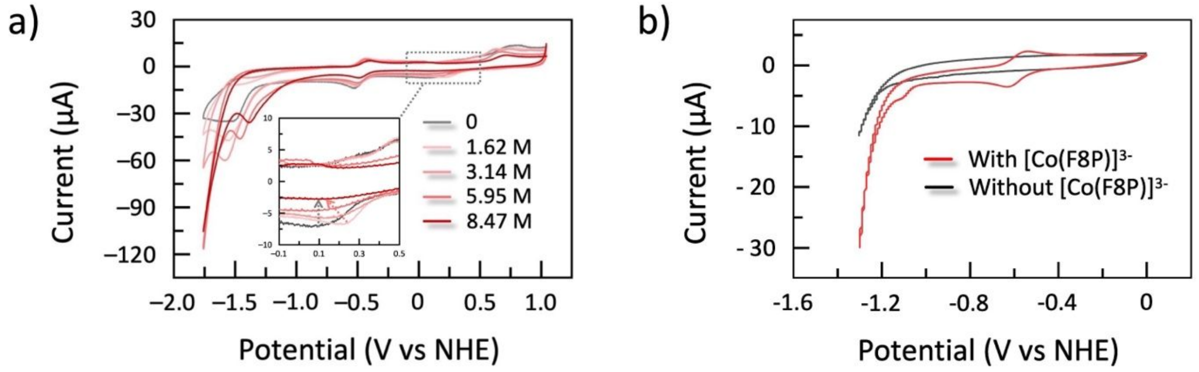

Figure S7 *Cyclic voltammetry of the [Co(F8P)]$^{3-}$* ***a)*** *in DMF with addition of 0 to 8.47 M water;* ***b)*** *in KCl aqueous solution. Conditions:* ***a)*** *1.0 mM Na_3[Co(F8P)], 0.1 M $TBAPF_6$, SCE reference electrode, 200 mV s^{-1} scan rate;* ***b)*** *0.5 mM Na_3[Co(F8P)], 0.1 M KCl, Ag/AgCl reference electrode, 50 mV s^{-1} scan rate. 0.07 cm^2 glassy-carbon working electrode, Pt wire auxiliary electrode, T = 298 K.*

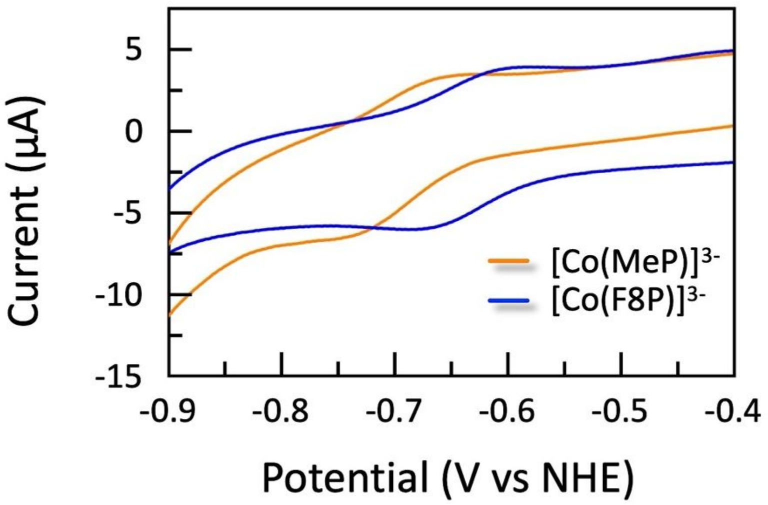

Figure S8 *Zoom of the cyclic voltammograms of [Co(MeP)]$^{3-}$ and [Co(F8P)]$^{3-}$ shown in Figure 3b from –0.9 to –0.4 V vs. NHE.*

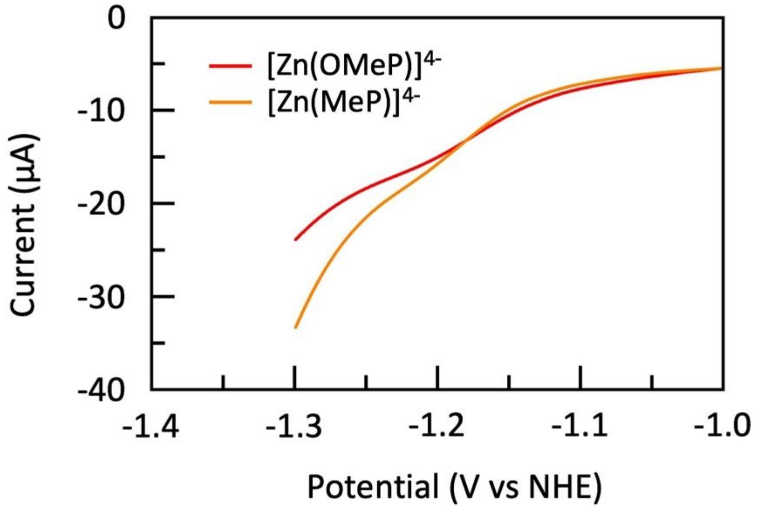

Figure S9 *Linear sweep voltammetry from –1.3 to –1.0 V vs. NHE of the [Zn(OMeP)]$^{4-}$ and [Zn(MeP)]$^{4-}$ in Figure 3c.*

Photocatalytic Hydrogen Evolution

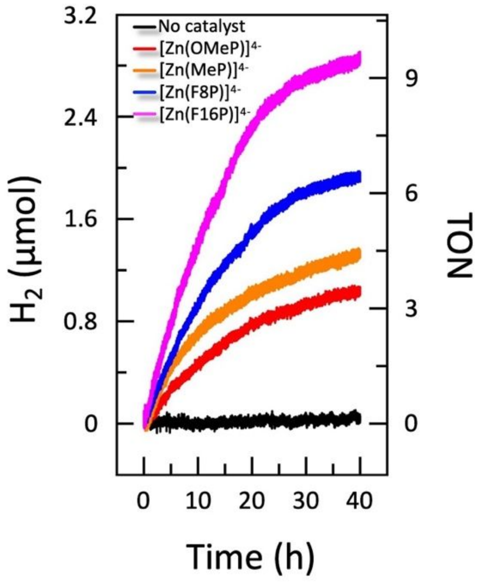

Figure S10 *Hydrogen evolution during photocatalytic water reduction in presence of 0.1 mM catalyst [Zn(OMeP)]$^{4-}$, [Zn(MeP)]$^{4-}$, [Zn(F8P)]$^{4-}$ and [Zn(F16P)]$^{4-}$, using 0.5 mM [Ru(bpy)$_3$]Cl$_2$ as photosensitizer, 0.1 M ascorbate and TCEP, in pH 7.0 aqueous solution, and LED lamp (450 nm, 16 mW) for irradiation, T = 298 K.*

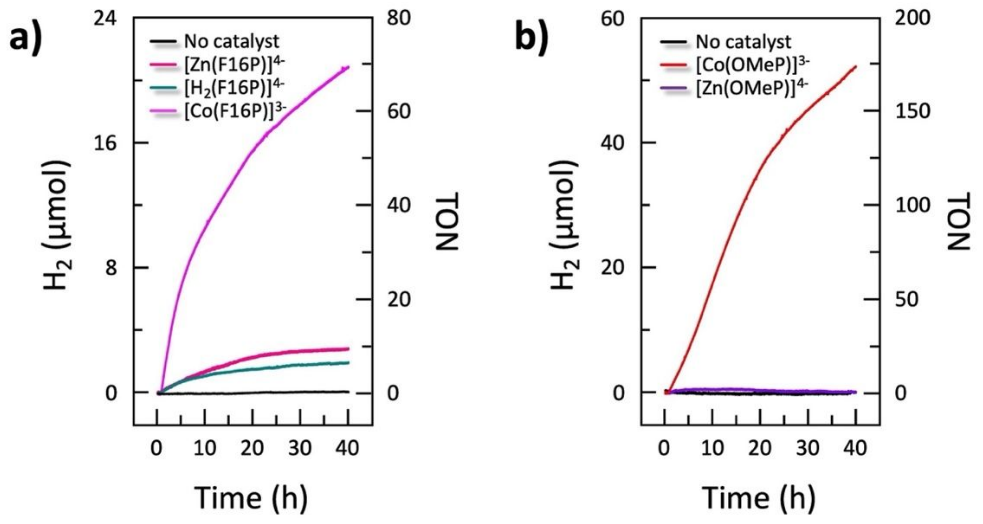

Figure S11 *Hydrogen evolution during photocatalytic water reduction in presence of a) 0.1 mM catalyst [Co(F16P)]$^{3-}$, [H$_2$(F16P)]$^{4-}$ and [Zn(F16P)]$^{4-}$ in pH 7.0 aqueous solution, and b) 0.1 mM catalyst [Co(OMeP)]$^{3-}$ and [Zn(OMeP)]$^{4-}$ in pH 4.1 aqueous solution using 0.5 mM [Ru(bpy)$_3$]Cl$_2$ as photosensitizer, 0.1 M ascorbate and TCEP, and LED lamp (450 nm, 16 mW) for irradiation, T = 298 K.*

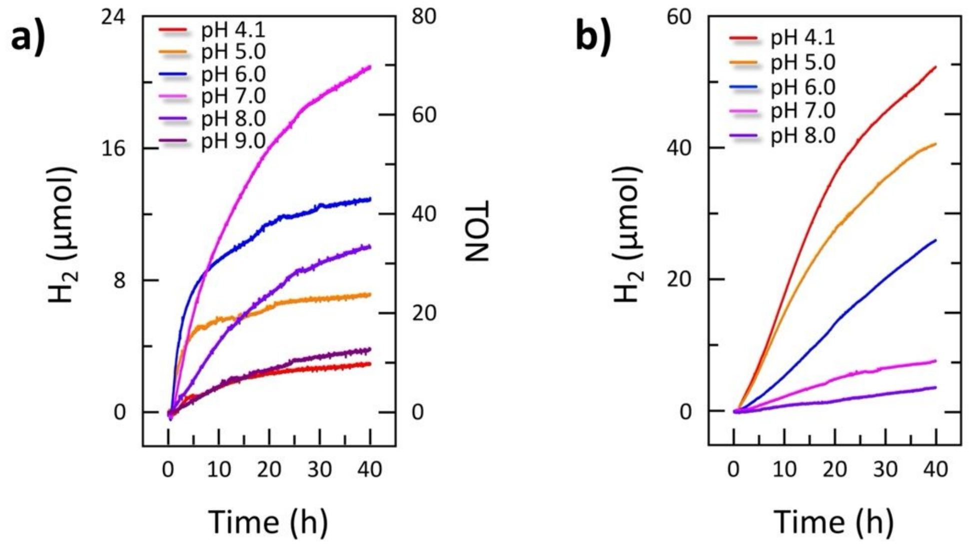

Figure S12 *Hydrogen evolution during photocatalytic water reduction in presence of a) 0.1 mM catalyst [Co(F16P)]*$^{3-}$ *and b) [Co(OMeP)]*$^{3-}$ *at different pH in aqueous solution using 0.5 mM [Ru(bpy)*$_3$*]Cl*$_2$ *as photosensitizer, 0.1 M ascorbic acid and TCEP as electron donor, and blue light irradiation (450 nm, 16 mW). Conditions: T = 298 K, phosphate buffer.*

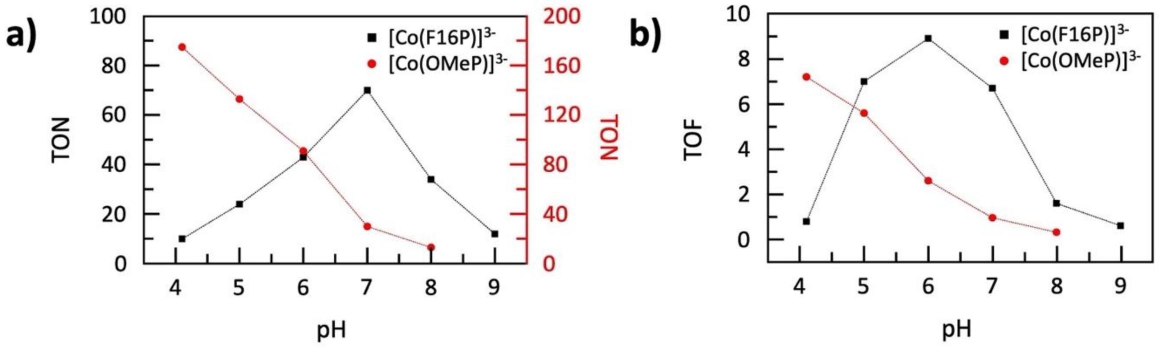

Figure S13 *a) Turnover numbers and b) maximum turnover frequencies of photocatalytic H*$_2$ *evolution systems in Figure S12 plotted as a function of the pH.*

Photostability

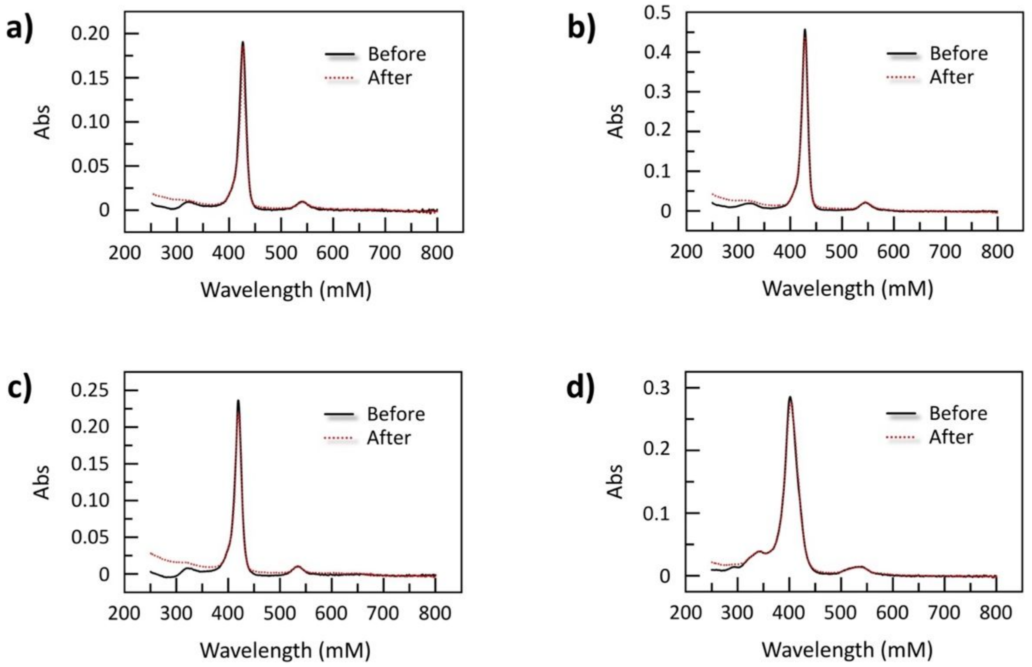

Figure S14 *Absorption spectra of 2 μM a) [Co(OMeP)]$^{3-}$, b) [Co(MeP)]$^{3-}$, c) [Co(F8P)]$^{3-}$, and d) [Co(F16P)]$^{3-}$ before (black solid) and after (red short dot) 48 hours irradiation (LED lamp, 450 nm, 16 mW) in 0.1 M sodium phosphate buffer (pH 7.0) under air, T = 298 K.*

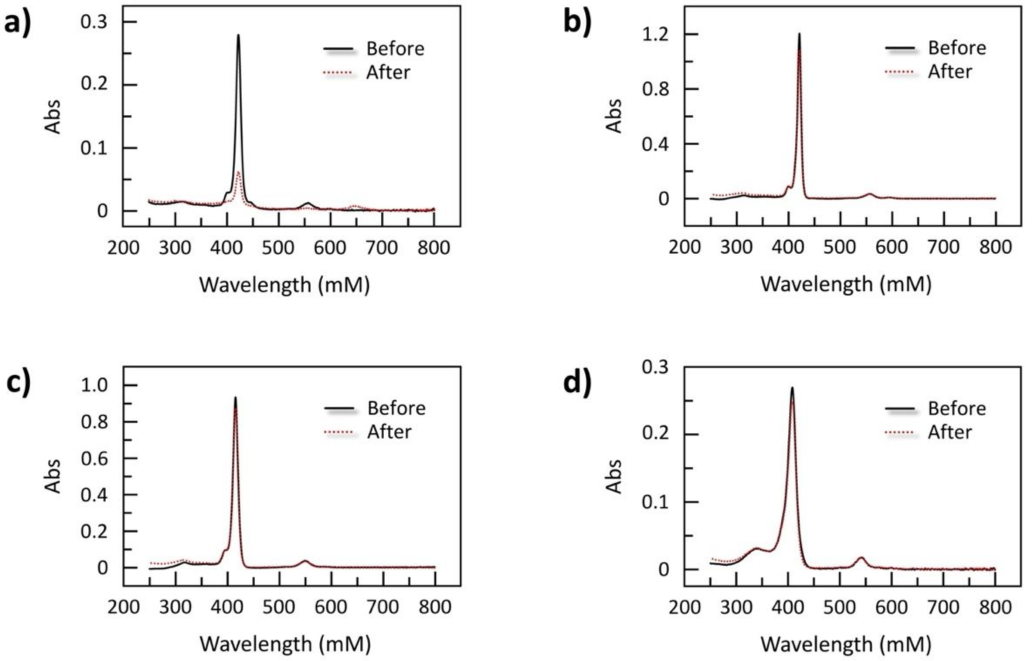

Figure S15 *Absorption spectra of 2 μM a) [Zn(OMeP)]$^{4-}$, b) [Zn(MeP)]$^{4-}$, c) [Zn(F8P)]$^{4-}$, and d) [Zn(F16P)]$^{4-}$ before (black solid) and after (red short dot) 48 hours irradiation (LED lamp, 450 nm, 16 mW) in 0.1 M sodium phosphate buffer (pH 7.0) under air, T = 298 K.*

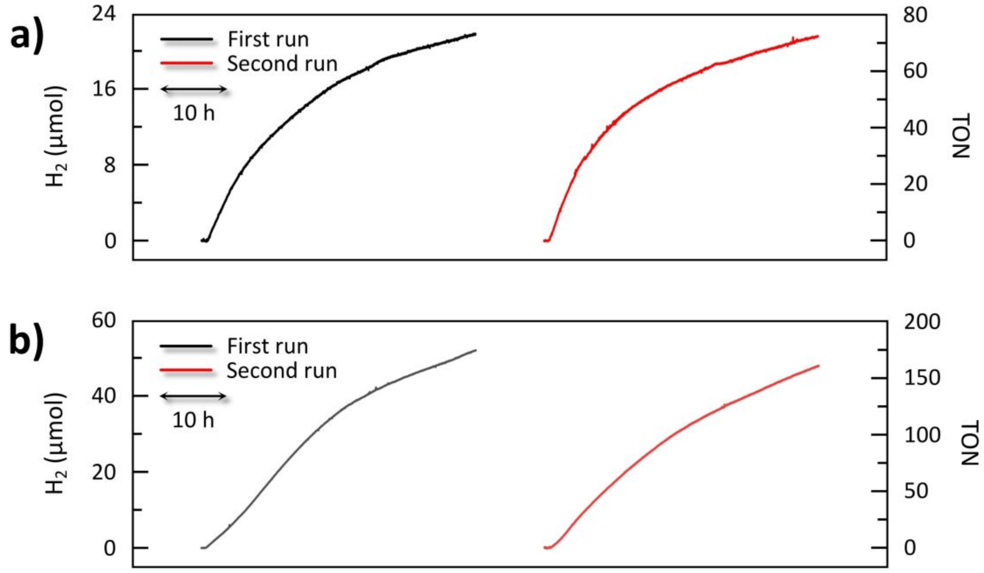

Figure S16 *Repetitive photocatalytic hydrogen evolution in presence of a) 0.1 mM [Co(F16P)]$^{3-}$ in pH 7.0 and b) [Co(OMeP)]$^{3-}$ in different pH 4.1 aqueous solution using 0.5 mM [Ru(bpy)$_3$]Cl$_2$ as photosensitizer, 0.1 M ascorbate and TCEP, and LED lamp (450 nm, 16 mW) for irradiation, T = 298 K. Between the two irradiation experiments, 1.5 μmol fresh [Ru(bpy)$_3$]Cl$_2$ was added as a solid.*

Kinetic Study

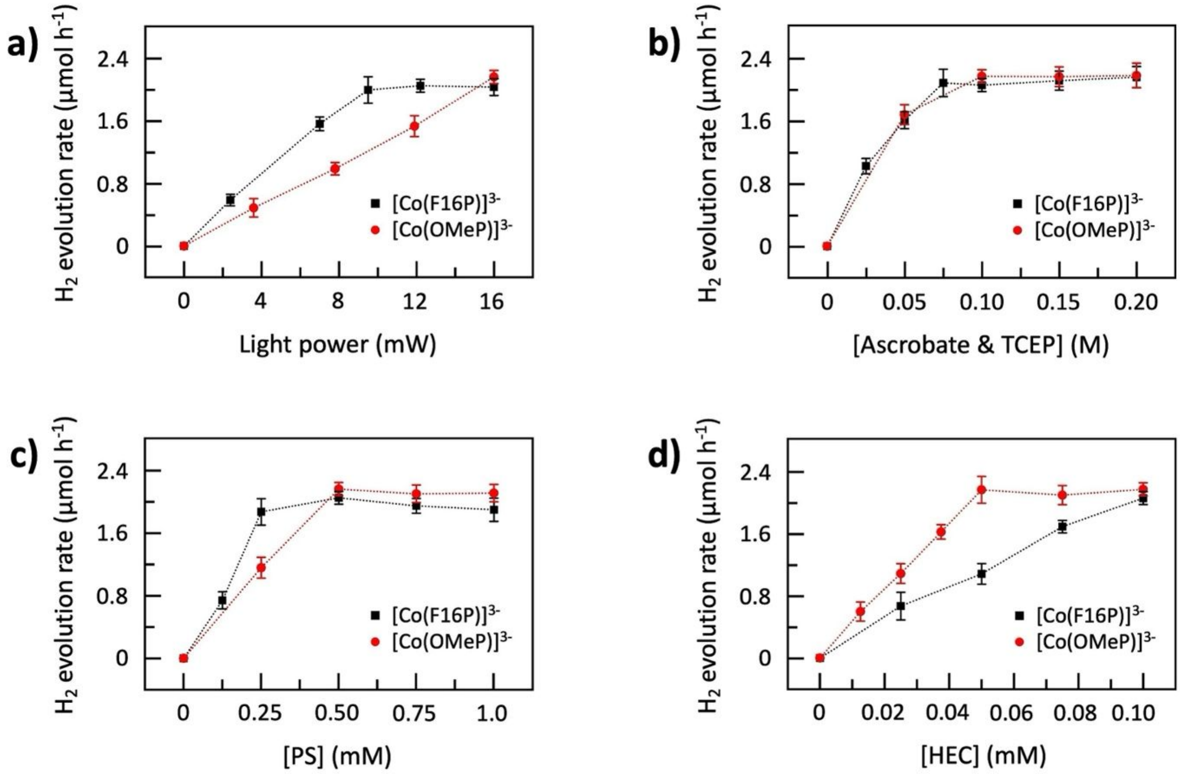

Figure S17 *Maximum H_2 evolution rate during photocatalytic H_2 evolution using the catalyst $[Co(F16P)]^{3-}$ at pH 7.0 (black squares) or $[Co(OMeP)]^{3-}$ at pH 4.1 (red circles) plotted as a function of a) the light power, b) the concentration of ascorbate and TCEP, c) the concentration of the photosensitizer $[Ru(bpy)_3]Cl_2$ and d) the concentration of the hydrogen evolution catalyst. Standard conditions: 0.1 mM catalyst, 0.5 mM $[Ru(bpy)_3]Cl_2$, 0.1 M ascorbate and TCEP, blue light (450 nm, 16 mW), T = 298 K.*

Density Functional Theory

Table S2 Gibbs free energies of all considered 1,2 and 3 electron reduced intermediates (in kcal/mol, relative to $^{2}[Co^{II}(P)]^{4-}$).[a]

	Relative energy (kcal mol^{-1})	
**Species**	**[Co(F16P)]**	**[Co(OMeP)]**
**1 e^{-} reduced**		
$^{2}[Co^{II}(P)]^{4-}$	0	0
$^{4}[Co^{II}(P)]^{4-}$	-	16.4
$^{2}[Co^{II}(P)(H_2O)]^{4-}$	3.5	1.7
$^{2}[Co^{II}(P)(H_2O)_2]^{4-}$	8.0	14.0
**2 e^{-} reduced**		
$^{1}[Co^{I}(P)]^{5-}$	12.1	16.5
$^{3}[Co^{I}(P)]^{5-}$	26.9	29.0
$^{3}[Co^{I}(P)(H_2O)]^{5-}$	26.6 [c]	29.9 [c]
$^{1}[Co^{III}(H)(P)]^{4-}$	14.5	10.5
$^{1}[Co^{III}(H)(P)(H_2O)]^{4-}$	19.8	13.7 [c]
$^{1}[Co^{I}(P\text{-}H_N)]^{4-}$	24.2	21.8
$^{1}[Co^{I}(P\text{-}H_\alpha)]^{4-}$	26.4	24.7
$^{1}[Co^{I}(P\text{-}H_\beta)]^{4-}$	21.4	16.4
$^{1}[Co^{I}(P\text{-}H_{meso})]^{4-}$	30.1	20.1
$^{3}[Co^{I}(P\text{-}H_\alpha)]^{4-}$	31.5[b]	22.6[b]
$^{3}[Co^{I}(P\text{-}H_\beta)]^{4-}$	25.6[b]	21.1
$^{3}[Co^{I}(P\text{-}H_{meso})]^{4-}$	21.9[b]	14.8
$^{3}[Co^{I}(P\text{-}H_\alpha)(H_2O)]^{4-}$	33.2	29.7

**$^{3}[Co^{I}(P\text{-}H_{\beta})(H_2O)]^{4-}$**	27.3	24.2
**$^{3}[Co^{I}(P\text{-}H_{meso})(H_2O)]^{4-}$**	24.6	21.9
**3 e$^-$ reduced**		
**$^{2}[Co^{0}(P)]^{6-}$**	45.3	57.2
**$^{2}[Co^{II}(H)(P)]^{5-}$**	35.2	40.8
**$^{2}[Co^{0}(P\text{-}H_{N})]^{5-}$**	43.3	52.9
**$^{2}[Co^{0}(P\text{-}H_{\alpha})]^{5-}$**	30.6	41.5
**$^{2}[Co^{0}(P\text{-}H_{\beta})]^{5-}$**	22.7	34.1
**$^{2}[Co^{0}(P\text{-}H_{meso})]^{5-}$**	31.1	30.2

[a] Gibbs free energies assume concentrations of 0.1mM for all metal complexes and 55 M for water. All Gibbs free energies are calculated at pH = 0, at a potential of 0 V vs NHE and a temperature of 298.15 K.

[b] Only a single-point calculation in the low-spin optimized geometries were performed, as a geometry optimizations of the high-spin state structures did not converge due to issues with the SCF procedure.

[c] Aqua ligands partially dissociated during optimizations.

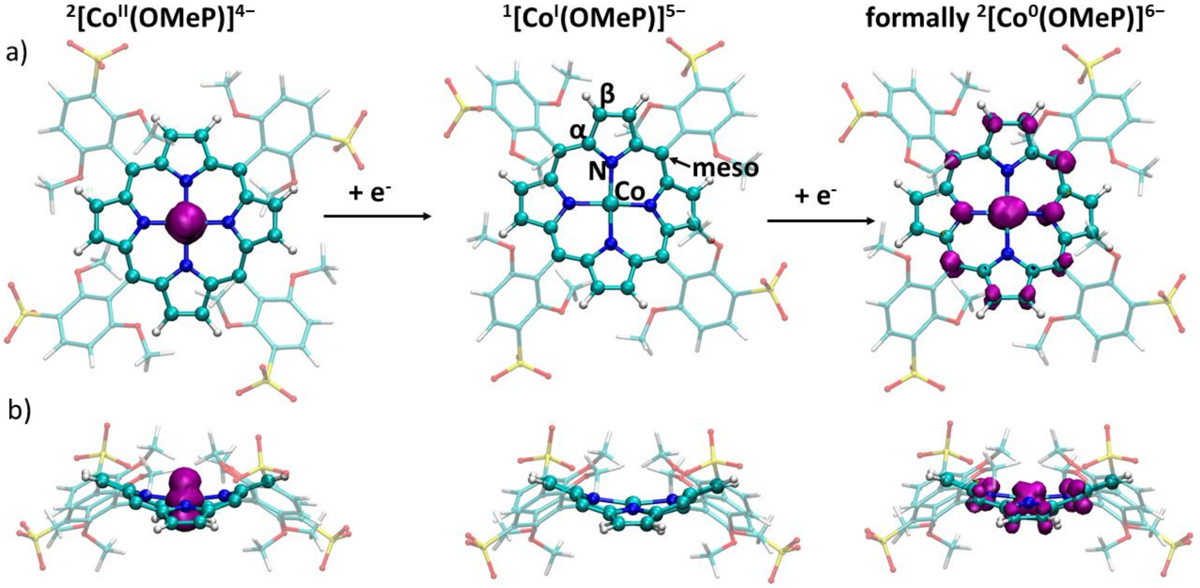

Figure S18 *Top (a) and side (b) views of the optimized structures of the [Co(OMeP)]$^{3-}$ catalyst complex in the Co^{II}, Co^I and formally Co^0 oxidation states. For the complexes with unpaired electrons, the spin density is visualized with an isosurface value of 0.003. The five different sites on the cobalt porphyrin complex which are prone to protonation are indicated with insets on the ^{1}[Co^I(OMeP)]$^{5-}$ intermediate.*

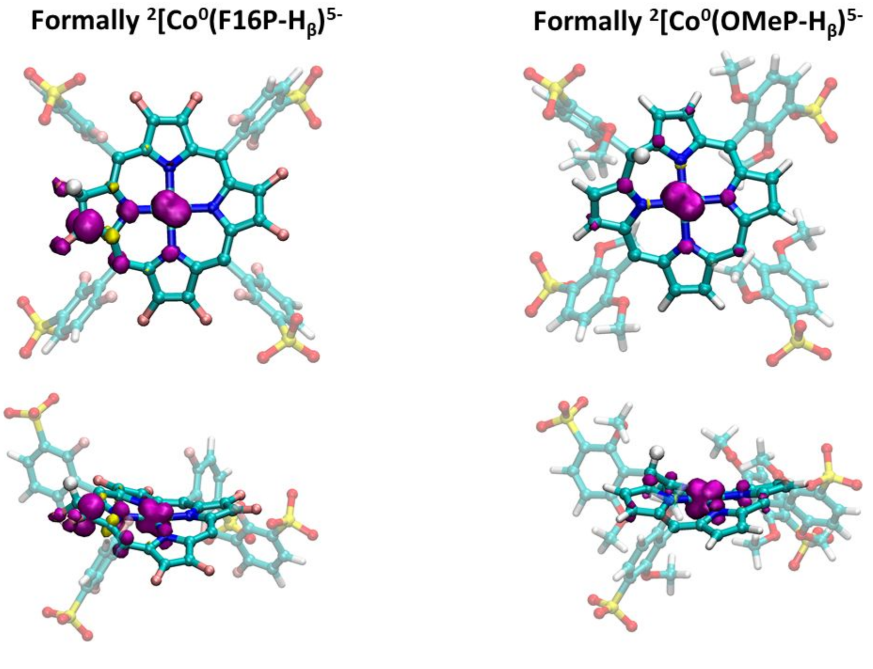

Figure S19 *Top and side views of the spin densities of the ^{2}[Co^0(F16P-H$_\beta$)]$^{5-}$ (b) and ^{2}[Co^0(OMeP-H$_{meso}$)]$^{5-}$ intermediates. Spin densities were computed at the PBE/def2TZVP/SMD level of theory and*

the isosurface value was set to 0.003. The hydride hydrogen atom is depicted with a VDW sphere with radius of 0.4, while all other atoms are visualized with radius of 0.2.

Table S3 Calculated one electron reduction potentials of the Co^{II} intermediates at the PBE/def2TZVP/SMD and OPBE/TZ2P/COSMO levels of theory.

**Redox couple**	**PBE/def2TZVP/SMD**	**OPBE/TZ2P/COSMO**
$^{2}[Co^{II}(F16P)]^{-4} \rightarrow {}^{1}[Co^{I}(F16P)]^{-5}$	–0.52	–0.50
$^{2}[Co^{II}(OMeP)]^{-4} \rightarrow {}^{1}[Co^{I}(OMeP)]^{-5}$	–0.70	–0.70

Table S4 Reduction potentials (in V vs. NHE) of the $^{2}[Co^{II}(F16P)]^{4-}$ to $^{1}[Co^{I}(F16P)]^{5-}$ reduction, calculated with six different exchange correlation functionals on the OPBE-D3BJ/ZORA-DZP geometries.

	OPBE	***PBE***	***wB97X***	***PBE0***	***B3LYP***	***M06[a]***	***Exp[B]***
**Reduction potential (V vs NHE)**	–0.50	–0.27	–1.11	–1.33	–0.98	–1.04	–0.53

[a] No dispersion corrections were included for the calculations with the M06 functional [b] data according to CV, V vs. NHE at pH 7 phosphate buffer as reported in this work.

Table S5 Calculated hydricity values for catalytic intermediates capable of H_2 generation.

Reaction	ΔG hydricity (kcal mol^{-1})
$^{2}[Co(II)(H)(F16P)]^{5-} \rightarrow {}^{2}[Co(II)(F16)]^{4-} + H^{-}$	7.8
$^{2}[Co(0)(F16P\text{-}H_{\alpha})]^{5-} \rightarrow {}^{2}[Co(II)(F16)]^{4-} + H^{-}$	12.4
$^{2}[Co(0)(F16P\text{-}H_{\beta})]^{5-} \rightarrow {}^{2}[Co(II)(F16)]^{4-} + H^{-}$	20.3
$^{2}[Co(0)(F16P\text{-}H_{meso}]^{5-} \rightarrow {}^{2}[Co(II)(F16)]^{4-} + H^{-}$	11.9
$^{2}[Co(0)(F16P\text{-}H_{N}]^{5-} \rightarrow {}^{2}[Co(II)(F16)]^{4-} + H^{-}$	−0.2
$^{2}[Co(II)(H)(OMe)]^{5-} \rightarrow {}^{2}[Co(II)(OMeP)]^{4-} + H^{-}$	2.3
$^{2}[Co(0)(OMe\text{-}H_{\alpha})]^{5-} \rightarrow {}^{2}[Co(II)(OMeP)]^{4-} + H^{-}$	1.5
$^{2}[Co(0)(OMe\text{-}H_{\beta})]^{5-} \rightarrow {}^{2}[Co(II)(OMeP)]^{4-} + H^{-}$	9.0
$^{2}[Co(0)(OMe\text{-}H_{meso}]^{5-} \rightarrow {}^{2}[Co(II)(OMeP)]^{4-} + H^{-}$	13.1
$^{2}[Co(0)(OMe\text{-}H_{N}]^{5-} \rightarrow {}^{2}[Co(II)(OMeP)]^{4-} + H^{-}$	−9.9

Table S6 Thermodynamic cycle employed for calculating hydricity values.

number	Reaction		value
Reaction 1	$D\text{-}H \rightarrow D^{+} + H^{-}$	→	
reaction 2	$D\text{-}H \rightarrow D^{-} + H^{+}$	→	DFT
reaction 3	$D^{+} + 2e^{-} \rightarrow D^{-}$	→	DFT
reaction 4	$H_2 \rightarrow H^{-} + H^{+}$	→	Reference[1]
reaction 5	$2H^{+} + 2e^{-} \rightarrow H_2$	→	DFT

ΔG(reaction 1) = ΔG(reaction 2) - ΔG(reaction 3) + ΔG(reaction 4) + ΔG(reaction 5).

Calculation of Maximum H_2 Evolution Rate and Maximum TOF

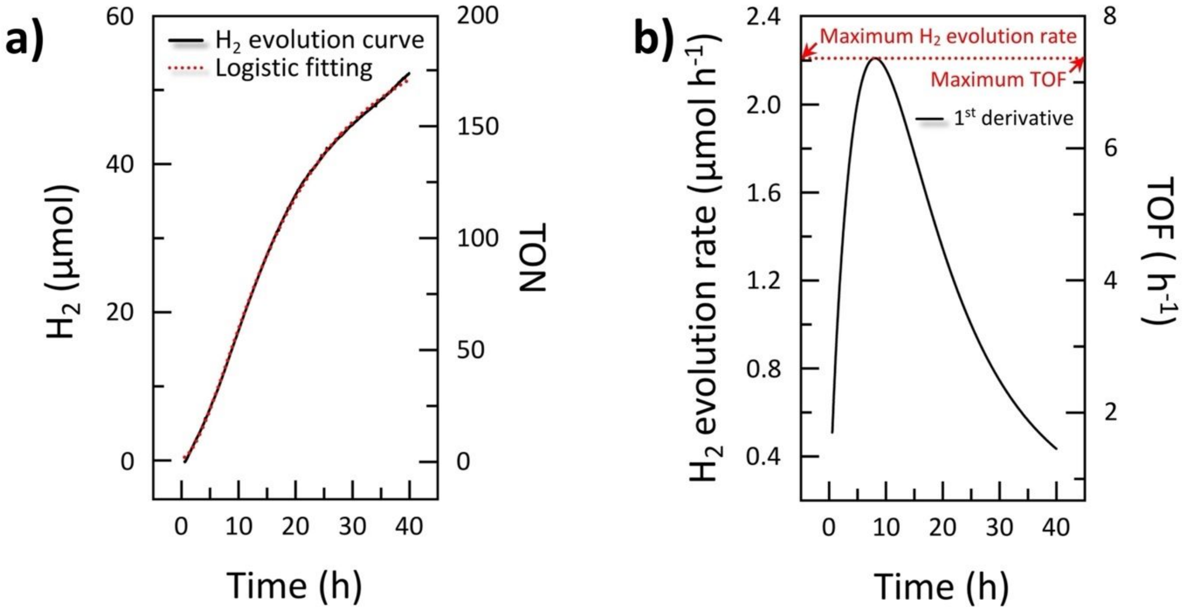

Figure S20 *Calculation of maximum H_2 evolution rate and maximum TOF of $[Co(OMeP)]^{3-}$.*

(1) Wiedner, E. S.; Chambers, M. B.; Pitman, C. L.; Bullock, R. M.; Miller, A. J. M.; Appel, A. M. Thermodynamic Hydricity of Transition Metal Hydrides. *Chem. Rev.* **2016**, *116* (15), 8655–8692. https://doi.org/10.1021/acs.chemrev.6b00168.
